# Neutrophil Extracellular Trap Reprograms Cancer Metabolism to Form a Metastatic Niche Promoting Non‐Small Cell Lung Cancer Brain Metastasis

**DOI:** 10.1002/advs.202508478

**Published:** 2025-11-18

**Authors:** Bo Chen, Karrie M. Kiang, Fangkun Liu, Chuntao Li, Xizhe Li, Chen Weiwei, Xin Fu, Gelei Xiao, Jingyi Sun, Erhan Da, Junbo Liao, Hongshu Zhou, Li Meng, Li Zhou, Tao Song, Longbo Zhang, Gilberto Ka‐Kit Leung, Liyang Zhang

**Affiliations:** ^1^ Department of Neurosurgery Xiangya Hospital Central South University 87 Xiangya Road Changsha Hunan 410008 China; ^2^ Department of Surgery School of Clinical Medicine LKS Faculty of Medicine The University of Hong Kong Queen Mary Hospital Hong Kong SAR China; ^3^ Department of Neurosurgery National Regional Center for Neurological Diseases Xiangya Hospital (Jiangxi) Central South University Nanchang Jiangxi 330038 China; ^4^ Hypothalamic Pituitary Research Center Xiangya Second Hospital Central South University 87 Xiangya Road Changsha Hunan 410008 China; ^5^ Department of Thoracic Surgery Xiangya Hospital Central South University 87 Xiangya Road Changsha Hunan 410008 China; ^6^ Department of Clinical Oncology School of Clinical Medicine LKS Faculty of Medicine The University of Hong Kong Queen Mary Hospital Hong Kong SAR China; ^7^ The State Key Laboratory of Ophthalmology Zhongshan Ophthalmic Center Sun Yat‐Sen University 54 Xianlie South Rd Guangzhou 510060 China; ^8^ Department of Respiratory Medicine Hunan Provincial People's Hospital (The First‐Affiliated Hospital of Hunan Normal University) 61 Jiefang Xi Road Changsha Hunan 410005 China; ^9^ Department of Radiology Xiangya Hospital Central South University 87 Xiangya Road Changsha Hunan 410008 China; ^10^ Department of Pathology Xiangya Hospital Central South University 87 Xiangya Road Changsha Hunan 410008 China; ^11^ National Clinical Research Center for Geriatric Disorders Xiangya Hospital Central South University Changsha Hunan China; ^12^ The State Key Laboratory of Brain and Cognitive Sciences The University of Hong Kong Hong Kong SAR China

**Keywords:** brain metastasis, metabolic reprogramming, metastasis‐initiating cells, neutrophil extracellular traps, non‐small cell lung cancer

## Abstract

Non‐small cell lung cancer (NSCLC) is the leading cause of brain metastases (BMs) and is characterized by a poor prognosis and limited response to standard treatments. Multi‐omics sequencings, integrating spatial transcriptomics, metabolomics, single‐cell RNA sequencing, bulk proteomics, and metabolomics, are conducted to analyze tumor and blood specimens from 34 patients with NSCLC with or without BMs from the Xiangya Hospital NSCLC (XY‐NSCLC) and Queen Mary Hospital NSCLC (QMH‐NSCLC) cohorts. This investigation identified *LOX* ^+^ Malig‐5 cells as metastasis‐initiating cells (MICs) that are significantly associated with poor prognosis. MICs colocalize with specific neutrophil subtypes, which facilitate the formation of neutrophil extracellular traps (NETs) within the metastatic niche. Mechanistically, a NET‐KRT10 signaling axis that mediates the interaction between NET‐releasing neutrophils and *LOX* ^+^ Malig‐5 cells is discovered, thereby promoting epithelial–mesenchymal transition (EMT) and metastasis. Furthermore, metabolic profiling reveals elevated palmitic acid levels in the resulting metastatic niche, which emerges as a crucial metabolic driver in BMs. Using an AI‐driven prediction model and in vitro/in vivo assays, fatty acid synthase inhibitor TVB‐2640 is identified as a potential therapeutic agent for disrupting metabolic vulnerability and suppressing NSCLC BMs. These findings provide novel insights into NET‐dependent cellular interactions that sustain the pro‐metastatic microenvironment underlying NSCLC BMs, offering robust development of novel metabolism‐based therapeutic strategies to combat this lethal complication.

## Graphical abstract

This study mainly combines spatial/single‐cell transcriptomics and metabolomics to reveal *LOX* ^+^ Malig‐5 as NSCLC brain metastasis‐initiating cells, forming a niche with NET‐releasing neutrophils via the NET‐KRT10 axis. The metastatic niche is involved with metabolic reprogramming, with palmitic acid serving as a key metabolite. Fatty acid synthase inhibitors are promising therapeutic agents targeting palmitic acid to inhibit NSCLC brain metastasis.

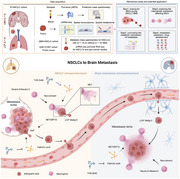

## 1 Introduction

Brain metastases (BMs), a common complication of non‐small cell lung cancer (NSCLC), occur in ≈40% of patients, with 10%–15% exhibiting radiographic evidence at initial diagnosis.^[^ ^1^, ^2^, ^3^ ^]^ Formation of a metastatic niche requires metastasis‐initiating cells (MICs) to undergo sequential changes, enabling epithelial–mesenchymal transition (EMT) and subsequent invasion. MICs are a rare subset of cancer cells with stem‐like properties and metabolic reprogramming that allows them to initiate and sustain metastasis.^[^ ^4^, ^5^ ^]^ Their presence is linked to metastatic recurrence and resistance to therapy, contributing to poor patient prognosis and increased mortality.^[^ ^6^ ^]^ Recent experimental studies have indicated that MICs rely on fatty acid metabolism and that sorting tumor cells based on lipid storage is crucial for identifying MICs in breast cancer.^[^ ^7^ ^]^ Additionally, palmitic acid supplementation enhances the metastatic potential of MICs in oral cancer.^[^ ^6^ ^]^

Early insights into classical “seed and soil” hypotheses underscore the importance of understanding MICs and their interactions with the tumor microenvironment.^[^ ^8^ ^]^ Neutrophils and their released extracellular structures, known as neutrophil extracellular traps (NETs),^[^ ^9^, ^10^ ^]^ play crucial roles in metastatic processes. NETs are comprised of granule‐derived lytic cationic antimicrobial peptides, proteases, and histones.^[^ ^11^ ^]^ MICs induce NET formation and facilitate metastasis through multiple mechanisms, including damage to endothelial cells, induction of angiogenesis, remodeling of the extracellular matrix, formation of physical scaffolds, regulation of vascular adhesion, alteration of the metabolic status, and promotion of EMT in tumor cells.^[^ ^12^ ^]^ Targeting NET formation to disrupt NET‐tumor cell niches has emerged as a potential treatment strategy to impede key metastatic cascades, including tumor invasion, hematogenous spread, and distant colonization.^[^ ^13^ ^]^ How the tumor microenvironment shapes the pro‐metastatic phenotype should be elucidated to develop effective strategies to prevent cancer progression.

This study aimed to define the specialized microenvironment formed by MICs and their interacting cells as a metastatic niche. More importantly, the distinct metabolic features of the metastatic niche that support tumor invasion and metastasis were investigated. Alterations in cancer metabolism have been implicated in driving tumor metastasis.^[^ ^14^, ^15^ ^]^ However, traditional omics techniques remain limited in capturing the metabolic heterogeneity within tumors and fail to fully elucidate the complex metabolic interactions between different cells in the metastatic niche. In this study, we employed a comprehensive multi‐omics approach, integrating spatial transcriptomics, metabolomics, single‐cell transcriptomics, bulk transcriptomics, proteomics, and metabolomics to characterize cellular interactions and metabolic heterogeneity in primary NSCLC and BM samples. Pseudotime analyses identified MICs and their colocalized NET‐releasing neutrophils. Multiplex immunofluorescence and pull‐down assays revealed direct MIC/NET interactions, whereas computational analyses defined metastatic niches based on MIC/NET abundance, uncovering potential metabolic reprogramming. Finally, drug screening was conducted to identify the key metabolites that drive metastatic niche formation and progression. This study elucidates the metastatic biology and promotes the development of targeted metabolic interventions, offering new therapeutic avenues for NSCLC and its aggressive BMs.

## 2 Results

### 2.1 Cellular and Molecular Characterization of Primary NSCLC and BM Tissues Using Combined Single‐Cell Transcriptomics, Spatial Transcriptomics, and Metabolomics Analyses

**Figure 1:**
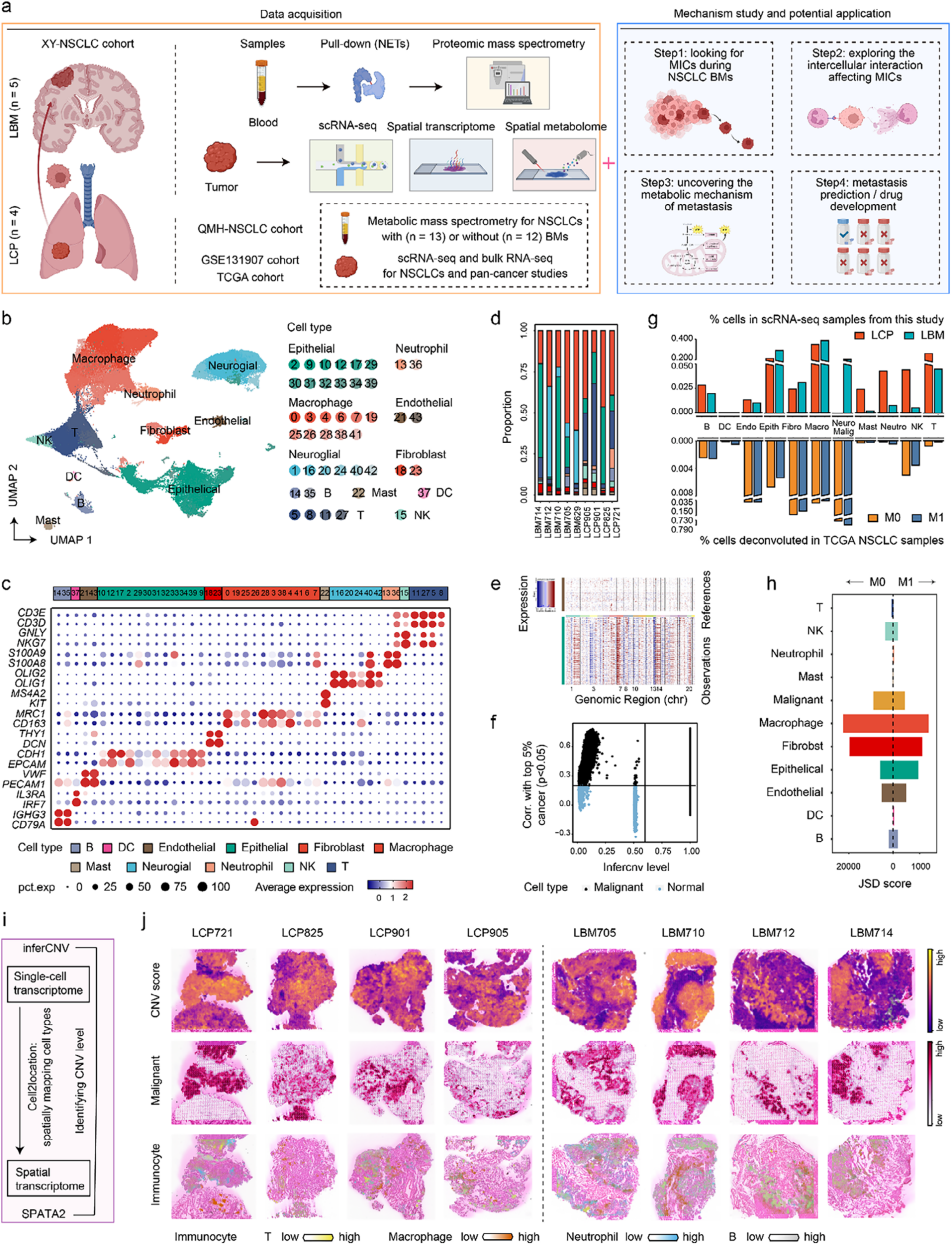
Cellular and molecular characterization of primary non‐small cell lung cancer (NSCLC) and brain metastasis (BM) tissues using combined single‐cell transcriptomics, spatial transcriptomics, and metabolomics analyses. a) Schematic diagram of data acquisition, mechanism study, and application exploration in this research. b) Uniform manifold approximation and projection (UMAP) plot showing the integrated single‐cell map from 4 lung cancer primary (LCP) and 5 lung cancer BM (LBM) lesions. Cells are colored by clusters. c) Dot plot displaying canonical marker genes across cell clusters. Dot size indicates the proportion of cells expressing the specific genes. Color intensity represents the average scaled expression level of specific genes. d) Stacked bar plot showing the proportions of annotated cell types across LCP and LBM samples. e) Hierarchical heatmap showing large‐scale copy number variations (CNVs) of epithelial cells to identify malignant cells. f) Classification of malignant cells and normal epithelial cells based on CNV levels and CNV correlation. g) Bar plots comparing cell type abundance between LCPs and LBMs based on our in‐house single‐cell RNA sequencing (scRNA‐seq) data (top), and comparing cell type abundance between LCPs without metastasis (M0) and LCPs with metastasis (M1) using deconvoluted bulk RNA‐seq data from the TCGA NSCLC cohort (bottom). h) Bar plot showing the heterogeneity of cell types among different samples from the TCGA NSCLC cohort based on Jensen–Shannon divergence (JSD) score. i) Schematic diagram illustrating spatial deconvolution using Cell2location and the evaluation of deconvolution accuracy with SPATA2. j) The spatial distributions of SPATA2‐calculated CNV scores (top), Cell2location‐deconvoluted malignant cells (middle), and immunocytes (bottom) across spatial transcriptomics slides from four LCPs and four LBMs. Schematic diagram (MIC, metastasis‐initiating cell; NET, neutrophil extracellular trap). Cell types (DC, dendritic cell; Endo, endothelial; Epith, epithelial cells; Fibro, fibroblast; Macro, macrophage; Malig, malignant; NK, natural killer; Neuro, neuroglial; Neutro, neutrophil).

To comprehensively characterize the cellular and metabolic profiles of NSCLC BMs, we utilized single‐cell transcriptomics, spatial transcriptomics, and spatial metabolomic sequencing to profile four lung cancer primary (LCP) and five lung cancer BM (LBM) tissues from patients in the Xiangya Hospital NSCLC (XY‐NSCLC) cohort (**Figure** **1**a, Table S1, Supporting Information). Single‐cell RNA sequencing (scRNA‐seq) analysis encompassed 94 383 cells categorized into 11 major cell types (Figure 1b,c; Figure S1a–c, Supporting Information), with immune cells comprising the largest proportion in both LCP (78.5%) and LBM (46.8%) (Figure 1d,g). InferCNV analysis further identified 21 396 malignant cells with high copy number variation (CNV) levels in epithelial cells (Figure 1e,f).

Next, we performed BayesPrism deconvolution analysis of the LCP scRNA‐seq to the Cancer Genome Atlas (TCGA) NSCLC bulk RNA‐seq data (Figure 1a). High homogeneity, indicated by a low Jensen–Shannon divergence (JSD) score, was observed in the composition of certain immune cells (e.g., neutrophils, DCs, and T cells) across specimens with the same metastasis status (Figure 1h). The proportion of neutrophils and DCs increased, whereas the abundance of T cells decreased in LCPs with metastasis compared to those without metastasis (Figure 1g). Immune cell subpopulation heterogeneity across the NSCLC metastasis stages is depicted in Figure S2 (Supporting Information), further highlighting the distinct cellular compositions of NSCLCs with and without metastasis.

Spatial information is crucial for understanding the tumor microenvironment. Accordingly, we performed Cell2location deconvolution analysis to locate the cellular components on eight spatial transcriptomic slides based on matched scRNA‐seq data (Figure 1i,j; Figure S1d, Supporting Information). The malignant cell distribution identified by the Cell2location closely matched the high‐CNV‐score regions calculated using SPATA2 (Figure 1j), confirming the accuracy of the deconvolution analysis. In addition, the distribution of malignant cells was related to the localization of immune cells (Figure 1j).

### 2.2 Differentiation Trajectories of Malignant Cells Defined the MIC Population

**Figure 2:**
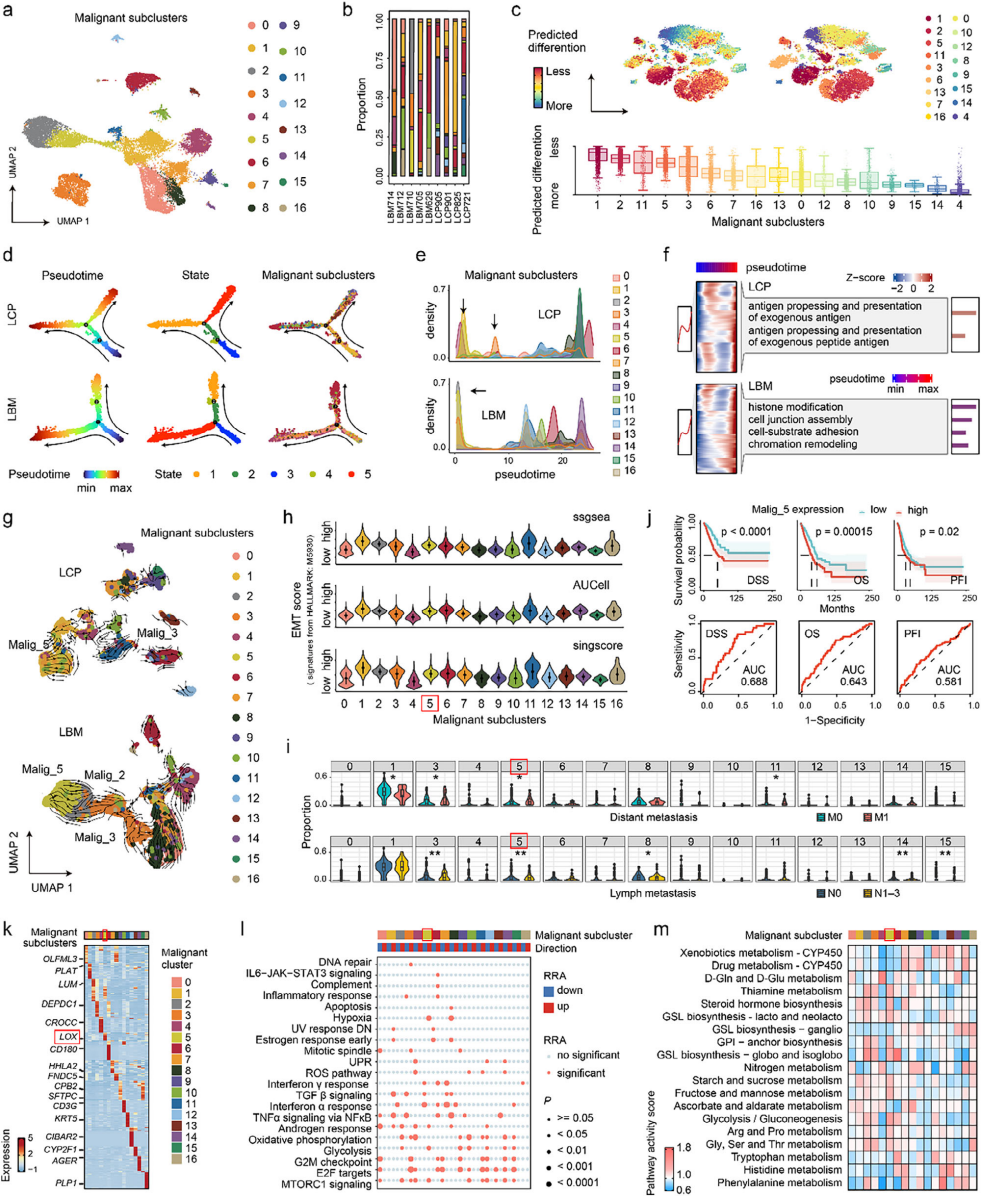
Differentiation trajectories of malignant cells define the MIC *LOX* ^+^ Malig‐5. a) UMAP plot showing 17 subclusters of malignant cells from LCPs and LBMs. Cells were colored by subclusters. b) Stacked bar plot showing the proportions of malignant cell subclusters across LCP and LBM samples. c) T‐distributed stochastic neighbor embedding (TSNE) plots (top) and bar plots (bottom) displaying differentiation states for each malignant cell subcluster using CytoTRACE. d) Monocle pseudotime trajectory of malignant cell subclusters in LCPs (top) and LBMs (bottom), inferred by Monocle2. Trajectories are colored by pseudotime (left), cell states (middle), and malignant cell subclusters (right). e) Cell density distribution of malignant cell subclusters along with the pseudotime in LCPs (top) and LBMs (bottom). Arrows refer to LBM differentiation‐initiating cells Malig‐2, Malig‐3, and Malig‐5. f) Heatmap displaying gene expression patterns of malignant cell subclusters and their gene ontology (GO) function enrichment along pseudotime in LCPs (top) and LBMs (bottom). g) VIA pseudotime trajectory model of malignant cell subclusters in LCPs (top) and LBMs (bottom), inferred by PyVIA. h) Violin plot showing epithelial‐mesenchymal transition (EMT) (HALLMARK: M5930) scores, quantified by ssGSEA (top), AUCell (middle), and singscore (bottom) for each malignant cell subcluster. The malignant cell subcluster circled in red boxes refers to the identified metastasis‐initiating cell (MIC), Malig‐5. i) Comparison of the proportion of malignant cell subclusters between lung cancers with metastasis (distant metastasis or lymph node metastasis) and without metastasis, as measured in the deconvoluted bulk RNA‐seq data from the TCGA NSCLC cohort. Two‐tailed Wilcoxon test. * *p* < 0.05, ** *p *< 0.01. j) Kaplan–Meier survival curves showing disease‐specific survival (DSS), overall survival (OS), and progression‐free interval (PFI) for the TCGA NSCLC cohort, stratified by the Malig‐5 proportion. Log‐rank test. k) Heatmap showing the expression of marker genes across malignant cell subclusters. Marker genes of Malig‐5 are highlighted within a red box. l) Heatmap displaying the distributions of significant hallmark gene sets identified through irGSEA across malignant cell subclusters. Up or down in the legend indicates whether the enrichment level of the gene sets in the subcluster is higher or lower compared to other subclusters. RRA, Robust rank aggregation. m) Heatmap visualizing Kyoto Encyclopedia of genes and genomes (KEGG) metabolic pathway activities, inferred from scRNA‐seq data, across malignant cell subclusters. Except where noted, *LOX* ^+^ Malig‐5 was highlighted by red boxes.

Transcriptional heterogeneity is increasingly recognized as a key factor in tumor initiation, progression, and metastasis. To identify the MICs, 21 396 malignant cells from the LCP and LBM were categorized into 17 subclusters, termed Malig_0 to Malig_16 (**Figure** **2**a,b; Figure S3a, Supporting Information). Cell lineage trajectories were investigated using five algorithms to identify MICs. CytoTRACE identified Malig‐1, Malig‐2, Malig‐11, Malig‐5, and Malig‐3 as highly undifferentiated cells with high stemness scores (Figure 2c). Monocle2 revealed that Malig‐2, Malig‐3, and Malig‐5 were differentiation‐initiating cells in LBMs. Only Malig‐3 and Malig‐5 were present in LCPs, with Malig‐5 positioned at the starting point and Malig‐3 appearing in the middle of the differentiation trajectories (Figure 2d,e). Trajectory analyses using three additional algorithms–the diffusion map, PyVIA, and RNA velocity–validated these findings and further indicated that Malig‐5 differentiates into Malig‐2, which subsequently produces Malig‐3 in LBMs (Figure 2g; Figure S3b–d, Supporting Information). Thus, Malig‐5 may be an MIC for NSCLC BMs. We further analyzed dynamic gene expression patterns along the differential trajectories of malignant cells. Gene ontology (GO) enrichment analysis showed that the terms related to antigen processing and presentation in LCPs and cell–cell junctions in LBMs were progressively activated along differential trajectories (Figure 2f; Figure S3e, Supporting Information). Malig‐5 cells may possess immune evasion, invasion, and migration capabilities. Among the 17 malignant cell subclusters, Malig‐5 also exhibited significantly higher levels of EMT process (Malig‐5 vs other subclusters, *p* < 0.001), ranking fifth on average, and markedly elevated hybrid‐EMT status (Malig‐5 vs other subclusters, *p* < 0.001), ranking fourth on average (Figure 2h; Figure S3f,g, Supporting Information).

Moreover, we investigated whether Malig‐5 exhibited a metastatic tendency and survival correlation in the TCGA NSCLC cohort. Bulk deconvolution data showed that NSCLC tumors with distant or lymph node metastases had a significantly higher proportion of Malig‐5 than those without (Figure 2i). NSCLC patients with high levels of Malig‐5s displayed significantly worse prognoses, including disease‐specific survival (DSS), overall survival (OS), and progression‐free interval (PFI) than those with low levels (Figure 2j). Among the 17 malignant cell subclusters, Malig‐5 demonstrated the highest predictive capability for metastasis and survival in patients with NSCLC, with an area under the curve (AUC) of 0.629 for distant metastasis, 0.589 for lymph node metastasis, 0.688 for DSS, 0.643 for OS, and 0.581 for PFI (Figure S3h,i, Supporting Information).

Using CellHint, we further annotated Malig‐5 in the publicly available scRNA‐seq validation dataset, which comprised 11 LCP and 10 LBM samples for a total of 74 209 cells (Figure S4a–c, Supporting Information). Consistent with the XY‐NSCLC data, Malig‐5 also exhibited high stemness, differentiation initiation potential, and elevated EMT scores in the validation set (Figure S4d–j, Supporting Information), supporting the hypothesis mentioned above that Malig‐5 may represent one of the MICs in NSCLC BMs.

### 2.3 MIC Malig‐5 is Characterized by a Unique Molecular Marker of LOX

To provide a more detailed characterization of the MIC (Malig‐5), we investigated its molecular profile, biological pathways, and metabolic traits within malignant cell subclusters. Differentially expressed gene (DEG) analysis revealed that lysyl oxidase (*LOX*) was uniquely and highly expressed in the Malig‐5 subcluster (avg_log2FC = 3.57, *p* < 0.001), ranking among the top six marker genes (Figure 2k; Figure S5a, Supporting Information). Correlation analysis revealed a strong positive association between *LOX* expression, EMT, and hybrid‐EMT status (Figure S5b, Supporting Information). This was also supported by the protein–protein interaction network, which showed a close interaction (interaction score > 0.2) between LOX and EMT proteins (Figure S5c, Supporting Information). Accordingly, we renamed the Malig‐5 cell sub‐cluster to provide more details and designated it *LOX* ^+^ Malig‐5. SCENIC analysis further revealed that EMT‐related transcription factors HOXA13,^[^ ^16^ ^]^ ONECUT2,^[^ ^17^ ^]^ and TFF3^[^ ^18^ ^]^ were highly expressed in *LOX* ^+^ Malig‐5 cells (Figure S5d, Supporting Information). Functional enrichment analysis of hallmark gene sets^[^ ^19^ ^]^ showed that the hypoxia and glycolysis pathways were significantly upregulated in *LOX* ^+^ Malig‐5 (Figure 2l), which was further confirmed by the quantification of metabolic pathway activity^[^ ^20^ ^]^ indicating that glycolysis/gluconeogenesis was a highly upregulated metabolic pathway in *LOX* ^+^ Malig‐5 (Figure 2m). Additionally, *LOX* ^+^ Malig‐5 cells exhibited a metabolically active state, featuring enhanced lipid metabolism compared to other malignant cell subclusters (Figure S5e, Supporting Information).

### 2.4 LOX+ Malig‐5s Colocalized with Neutrophils

**Figure 3:**
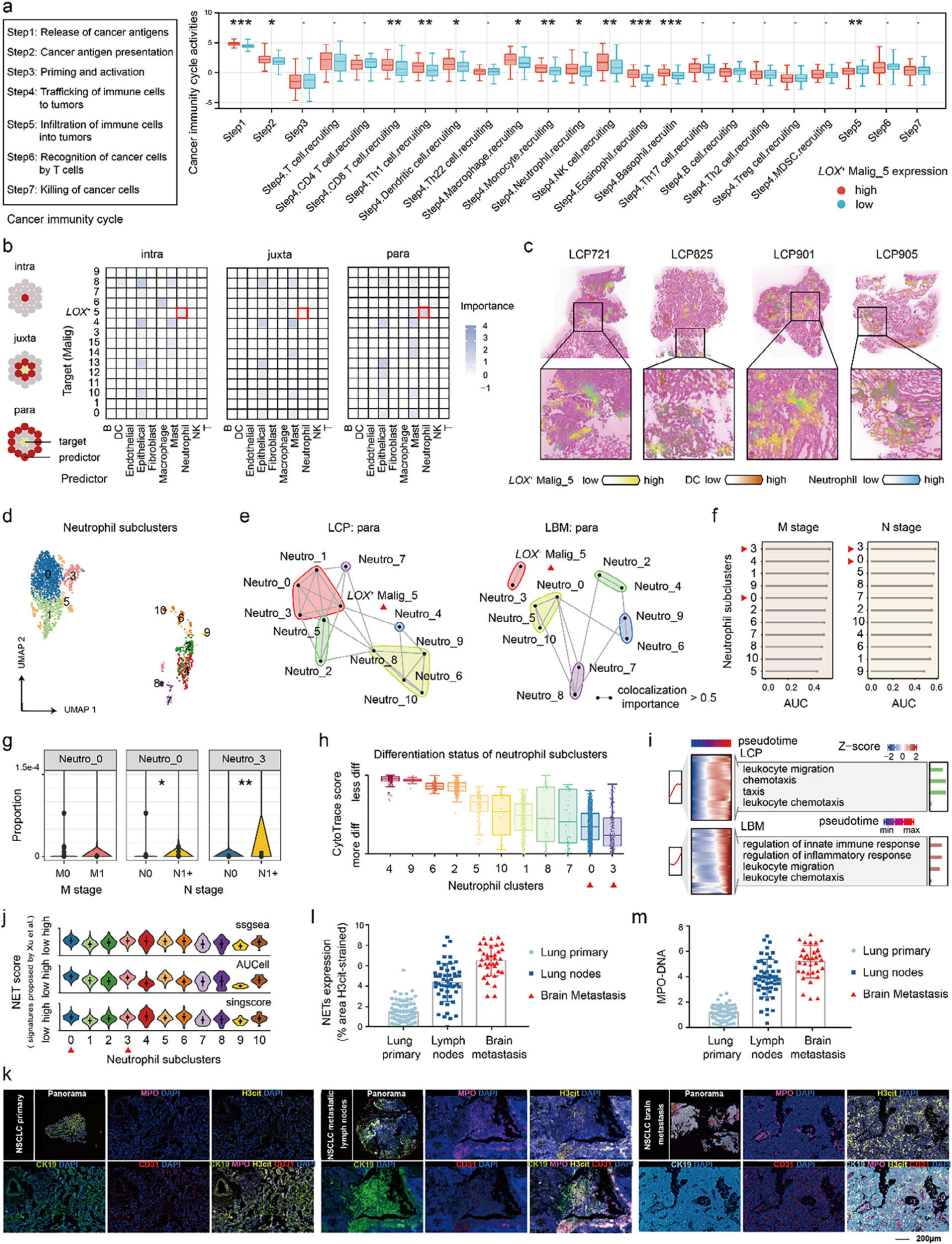
*LOX* ^+^ Malig‐5 colocalizes with the neutrophils, particularly with the subclusters Neutro‐0 and Neutro‐3. a) Comparisons of cancer immunity cycle activities between high and low *LOX* ^+^ Malig‐5 NSCLCs, as measured in the deconvoluted bulk RNA‐seq data from the TCGA cohort. Two‐tailed Wilcoxon test. * *p* < 0.05, ** *p* < 0.01, *** *p* < 0.001. b) The importance of cell colocalization across multiple neighborhood sizes (intra: within a spot, juxta: adjacent spot, and para: extending to two spots), as measured in the LCP spatial transcriptomic data. The cells circled in red boxes are neutrophils that are highly colocalized with *LOX* ^+^ Malig‐5. c) Visualization of the spatial distribution of *LOX* ^+^ Malig‐5 and neutrophils on LCP spatial transcriptomic slides using Cell2location. d) UMAP plot showing the single‐cell map of neutrophils, categorized into 11 subclusters. Cells were colored by neutrophil subclusters. e) Network community plots showing the colocalization relationship between *LOX* ^+^ Malig‐5 and neutrophil subclusters in the para view of LCP and LBM spatial transcriptomics slides. The lines connecting cells represent strong colocalizations, with an importance of >0.5. f) Ranking of neutrophil subclusters' ability with area under the curve (AUC) values to predict distant or lymphatic metastasis in the TCGA NSCLC cohort. The red triangle marks neutrophil subclusters Neutro‐0 and Neutro‐3 that show a strong correlation with metastasis and are highly colocalized with *LOX* ^+^ Malig‐5. g) Comparison of the proportions of Neutro‐0 and Neutro‐3 between NSCLCs with metastasis (distant or lymph node metastases) and without metastasis in the TCGA NSCLC cohort. Two‐tailed Wilcoxon test. * *p* < 0.05, ** *p* < 0.01. h) Bar plots displaying differentiation states for each neutrophil subcluster using CytoTRACE. i) Heatmap displaying dynamic gene expression patterns of neutrophil subclusters and their GO function enrichment along pseudotime in LCPs (top) and LBMs (bottom). j) Violin plot showing neutrophil extracellular trap (NET) release scores (signatures proposed by Xu et al.^[^ ^22^ ^]^), quantified by ssGSEA (top), AUCell (middle), and singscore (bottom) for each neutrophil subcluster in scRNA‐seq data. k) Multiplex immunofluorescence staining showing spatial locations of NETs (H3cit^+^ and MPO^+^), malignant cells (CK19^+^), and vascular endothelial cells (CD31^+^) in NSCLC primary (left), metastatic lymph node (middle), and BM (right) specimens. Scale bars, 800 µm (leftmost) and 200 µm (right). l,m) The bar plots show the quantification of NET expression (H3cit^+^, l; MPO^+^, m) between NSCLC primary, metastatic lymph node, and BM sites. Except where noted, *LOX* ^+^ Malig‐5, Neutro‐0, and Neutro‐3 were highlighted by red boxes or triangles.

The tumor immune microenvironment significantly influences every stage of metastasis. Analysis of TCGA bulk deconvolution showed that most immune co‐stimulatory genes were downregulated in high *LOX* ^+^ Malig‐5 NSCLCs compared to low *LOX* ^+^ Malig‐5 NSCLCs, whereas the majority of immune co‐inhibitors were upregulated (Figure S6a, Supporting Information), suggesting a potential immunosuppressive microenvironment in high *LOX* ^+^ Malig‐5 NSCLCs. This interesting finding is also supported by TCGA cancer immune cycle analysis, which showed that the infiltration of immune cells into tumors (step 5) was significantly downregulated in high *LOX* ^+^ Malig‐5 NSCLCs compared to that in low *LOX* ^+^ Malig‐5 NSCLCs (**Figure** **3**a).

Furthermore, we quantified *LOX* ^+^ Malig‐5 abundance in NSCLC bulk tissues and mapped its distribution in tumor sections. The proportion of *LOX* ^+^ Malig‐5s was negatively correlated with most immune cell proportions, but was positively associated with the neutrophil proportion in TCGA NSCLCs (Figure S6b, Supporting Information). TCGA cancer immune cycle analysis also revealed that neutrophil recruitment (step 4) was significantly increased in high *LOX* ^+^ Malig‐5 NSCLCs compared to that in low *LOX* ^+^ Malig‐5 NSCLCs (Figure 3a). Analysis of spatial transcriptomic data showed that neutrophils, among all immune and stromal cells in LCPs, exhibited the strongest spatial colocalization with *LOX* ^+^ Malig‐5 across multiple neighborhood sizes (intra: within a spot, juxta: adjacent spot, and para: extended to two spots) (Figure 3b,c; Figure S6c, Supporting Information). In LBMs, *LOX* ^+^ Malig‐5s also showed a high colocalization level with neutrophils (importance > 0.5) (Figure S6d,e, Supporting Information). Given that previous studies have highlighted the critical role of neutrophils in solid cancer metastases,^[^ ^21^ ^]^ we selected neutrophils as our research focus.

### 2.5 LOX^+^ Malig‐5s Particularly Colocalize with the Neutrophil Subclusters Neutro‐0s and Neutro‐3s

To further elucidate the detailed subclusters of neutrophils and their roles in the metastasis of *LOX* ^+^ Malig‐5s, we classified 2540 neutrophils into 11 subclusters, ranging from Neutro‐0 to Neutro‐10 (Figure 3d; Figure S7a–c, Supporting Information). Spatial colocalization analysis revealed that Neutro‐0s and Neutro‐3s strongly colocalized with *LOX* ^+^ Malig‐5s in LCPs and LBMs across multiple neighborhood sizes (intra, juxta, and para) (importance > 0.5) (Figure 3e; Figure S7d,e, Supporting Information). Bulk deconvolution analysis showed that NSCLCs with distant or lymphatic metastasis had a significantly higher proportion of Neutro‐0s and Neutro‐3 than those without metastasis (Figure 3g), with the metastasis prediction ability of Neutro‐0 and Neutro‐3 ranking at the top (1/11 for Neutro‐3, 2/11, and 5/11 for Neutro‐0) (Figure 3f). Neutro‐0s and Neutro‐3s may promote the metastasis of *LOX* ^+^ Malig‐5s in NSCLCs.

Next, we investigated the molecular, pathway, and evolutionary traits of Neutro‐0s and Neutro‐3s within the neutrophil subclusters. DEG and SCENIC analyses identified genes *PROK2 and ZBTB20*, along with the transcription factors STAT5B and RFX3, as molecular markers for Neutro‐0s and Neutro‐3s (Figure S8a,b, Supporting Information). Pseudo‐time analyses revealed the differentiation paths of neutrophils, ending with Neutro‐0s and Neutro‐3s, which are highly differentiated cells with low stemness (Figure 3h; Figure S8c–e, Supporting Information). During differentiation, the activation of leukocyte chemotaxis and migration‐related GO terms indicated robust chemotactic activity in both Neutro‐0s and Neutro‐3s (Figure 3i). Moreover, the metabolic pathway activity analysis suggested that both Neutro‐0s and Neutro‐3s were in a metabolically inactive state, except for fatty acid biosynthesis (Figure S8f,g, Supporting Information).

### 2.6 Neutro‐0s and Neutro‐3s as Pro‐Metastasis NET‐Releasing Neutrophils

NET release is a key characteristic of neutrophils and promotes cancer metastasis. Immunofluorescence analysis showed that NETs (H3cit^+^/MPO‐DNA^+^) colocalized with malignant cells (CK19^+^) in NSCLCs (Figure 3k). NET release was highest in BM sites, followed by lymph nodes, and lowest in primary lung sites (Figure 3l,m), suggesting the potential pro‐metastatic effect of NETs. Next, we quantified NET levels across neutrophil subclusters based on a gene set derived from a previous study.^[^ ^22^ ^]^ Among them, Neutro‐0s and Neutro‐3s exhibited the highest levels of NET release (Neutro‐0s/Neutro‐3s vs other sub‐clusters, *p* < 0.001), ranking first and second on average, respectively (Figure 3j). Subsequently, we calculated the within‐class neighborhood^[^ ^23^ ^]^ between the Malig‐5s and Neutro‐0s/Neutro‐3s. High‐NET regions, along with high‐EMT areas, were predominantly localized within the neighborhood formed by Malig‐5s and Neutro‐0s/Neutro‐3s (Figure S9a, Supporting Information). Spatial colocalization analysis also revealed strong colocalization between Neutro‐0s/Neutro‐3 and NET release, as well as between *LOX* ^+^ Malig‐5 and EMT, across multiple neighborhood sizes (intra, juxta, and para) (importance > 0.5) (Figure S9b, Supporting Information). Overall, Neutro‐0s and Neutro‐3s were confirmed as pro‐metastatic NET‐releasing neutrophils in NSCLCs.

### 2.7 Neutro‐0s and Neutro‐3s Interacted with LOX^+^ Malig‐5s through NET‐KRT10

**Figure 4:**
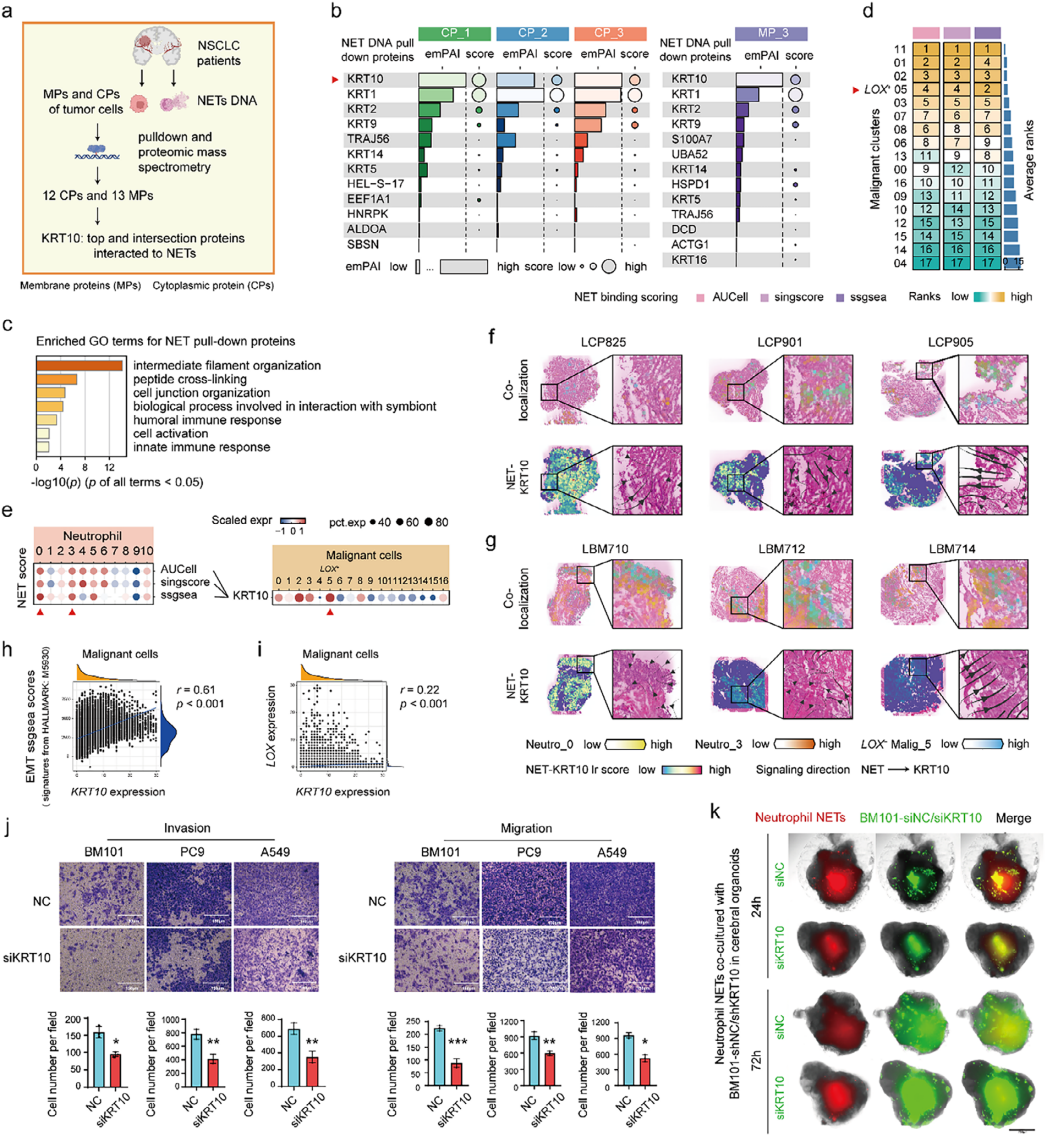
Neutro‐0 and Neutro‐3 are pro‐metastasis NETs releasing neutrophils, and interact with *LOX* ^+^ Malig‐5 through NET‐KRT10. a) Workflow of pull‐down assays and subsequent proteomic mass spectrometry sequencing analysis to identify NET‐binding proteins in NSCLC tumor cells. b) The emPAI values and scores for the top 12 cytoplasmic proteins (CPs) and 13 membrane proteins (MPs) from NSCLC tumor cells that bind to neutrophil NETs DNA. Among them, KRT10 (marked by a red triangle) ranked highest in both CPs and MPs. c) GO enrichment analysis of NET pull‐down proteins (including the top 12 CPs and 13 MPs) based on Metascape. d) Ranks of NET binding score (using the NET pull‐down proteins as a signature) of malignant cell subclusters in each quantification algorithm of AUCell, singscore, and ssGSEA. e) Cell–cell communication plots of NET‐KRT10 between neutrophil subclusters and malignant cell subclusters in the scRNA‐seq data. f,g) Visualization of the spatial distribution of Neutro‐0, Neutro‐3, and *LOX* ^+^ Malig‐5 using Cell2location (top), and signaling scores and direction of NET‐KRT10 using stLearn and COMMOT separately (bottom) on LCP (f) and LBM (g) spatial transcriptomics slides. h) Correlation between EMT ssGSEA scores and *KRT10* expression in malignant cells from scRNA‐seq data. i) Correlation between *LOX* and *KRT10* expression in malignant cells from scRNA‐seq data. Spearman correlation. j) Representative images (top) and quantification (bottom) of Transwell invasion (left) and migration (right) assays in BM101, PC9, and A549 cells with KRT10 siRNA or control siRNA transfection. Data are represented as means ± SEM of three independent experiments. Two‐tailed Wilcoxon test. * *p* < 0.05, ** *p* < 0.01, *** *p* < 0.001. k) BM101 cells (green fluorescence) and neutrophil NETs (red fluorescence) were transplanted into cerebral organoids. Following 24‐ and 72‐hour co‐culture periods, BM101 cells transfected with KRT10 siRNA or control siRNA exhibited distinct spreading rates within the fused organoids. Except where noted, *LOX* ^+^ Malig‐5, Neutro‐0, and Neutro‐3were highlighted by red triangles. Expr, Expression; Lr, Ligand‐receptor.

The spatial colocalization of Neutro‐0s/Neutro‐3s and *LOX* ^+^ Malig‐5 indicated their possible cell–cell interactions. Pull‐down assays and intercellular interaction analyses were performed to identify these interactions. We collected neutrophils from the blood of patients with NSCLC, induced NET release, and extracted tumor cell cytosol and membrane proteins from A549, BM101, and BM104 cells. Cytosolic and membrane proteins were pulled down using biotin‐labeled NET DNA, followed by proteomic mass spectrometry (**Figure** **4**a). Mass spectrometry‌ sequencing analysis identified 12 top cytoplasmic proteins (emPAI‐value > 0.1, score > 65.3) and 13 top membrane proteins (emPAI‐value > 1.1, score > 73) from tumor cells that bind to neutrophil NET DNA (Figure 4a,b; Figure S10a, Supporting Information). Functional enrichment analysis showed that these proteins were significantly enriched in GO terms related to cell junction organization and humoral immune response (*p* < 0.05) (Figure 4c), confirming their role as NET‐binding proteins in tumor cells. Further functional quantification analysis using these proteins as a signature revealed a significantly higher NET‐binding capacity of *LOX* ^+^ Malig‐5s (Malig‐5 vs other subclusters, *p* < 0.001), ranking top four among 17 malignant cell subclusters (Figure 4d; Figure S10b, Supporting Information).

KRT10, a type I intermediate filament protein, ranked first among both NET‐binding cytoplasmic and membrane proteins in NSCLC tumor cells (Figure 4b). Accordingly, we incorporated NET–KRT10 into ligand–receptor databases for multiple cell–cell interaction algorithms. CellChat analysis revealed that *LOX* ^+^ Malig‐5s could receive signals from Neutro‐0s and Neutro‐3s via NET–KRT10 communication at the single‐cell level (Figure 4e; Figure S10c, Supporting Information). Moreover, stLearn and COMMOT spatial interaction analyses, which illustrated that NET–KRT10 hotspots were primarily colocalized within the regions where Malig‐5s and Neutro‐0s/Neutro‐3s overlapped and that NET–KRT10 signals mainly flowed from Neutro‐0/Neutro‐3s to Malig‐5s (Figure 4f,g). We then examined KRT10's role in the invasion and metastasis of NSCLC cells. Among the top NET‐binding proteins, scRNA‐seq analysis showed that only *KRT10* expression was strongly correlated with EMT scores and *LOX* expression in malignant cells (*r* > 0.2) (Figure 4h,i; Figure S10d,e, Supporting Information). Transwell assays revealed significantly decreased invasion and migration following KRT10 knockdown in BM101, PC9, and A549 cells (Figure 4j). Furthermore, we co‐cultured BM101 cells with neutrophils in cerebral organoids and observed that KRT10 knockdown inhibited the spread of NSCLC cells in NET conditions (Figure 4k). We then investigated cell–cell communication from *LOX* ^+^ Malig‐5s to Neutro‐0s and Neutro‐3s using the same approach mentioned above. CellChat interaction analysis showed that *LOX* ^+^ Malig‐5s could send signals to Neutro‐0s and Neutro‐3s, primarily via CXCL8–CXCR2 at the single‐cell dimension (Figure S11a, Supporting Information). stLearn and COMMOT spatial interaction analyses further showed that CXCL8–CXCR2 hotspots were primarily colocalized within the regions where Malig‐5s and Neutro‐0s/Neutro‐3s overlapped, and the CXCL8–CXCR2 signal mainly flowed from Malig‐5s to Neutro‐0/Neutro‐3s (Figure S11b, Supporting Information). Overall, *LOX* ^+^ Malig‐5s could recruit Neutro‐0s and Neutro‐3s via the CXCL8–CXCR2 pathway. Consequently, Neutro‐0s and Neutro‐3s could release NETs that promote the EMT and metastasis of *LOX* ^+^ Malig‐5s by binding to KRT10.

### 2.8 Neutro‐0s, Neutro‐3s, and LOX^+^ Malig‐5s Form a Metastatic Niche with Metabolic Reprogramming

**Figure 5:**
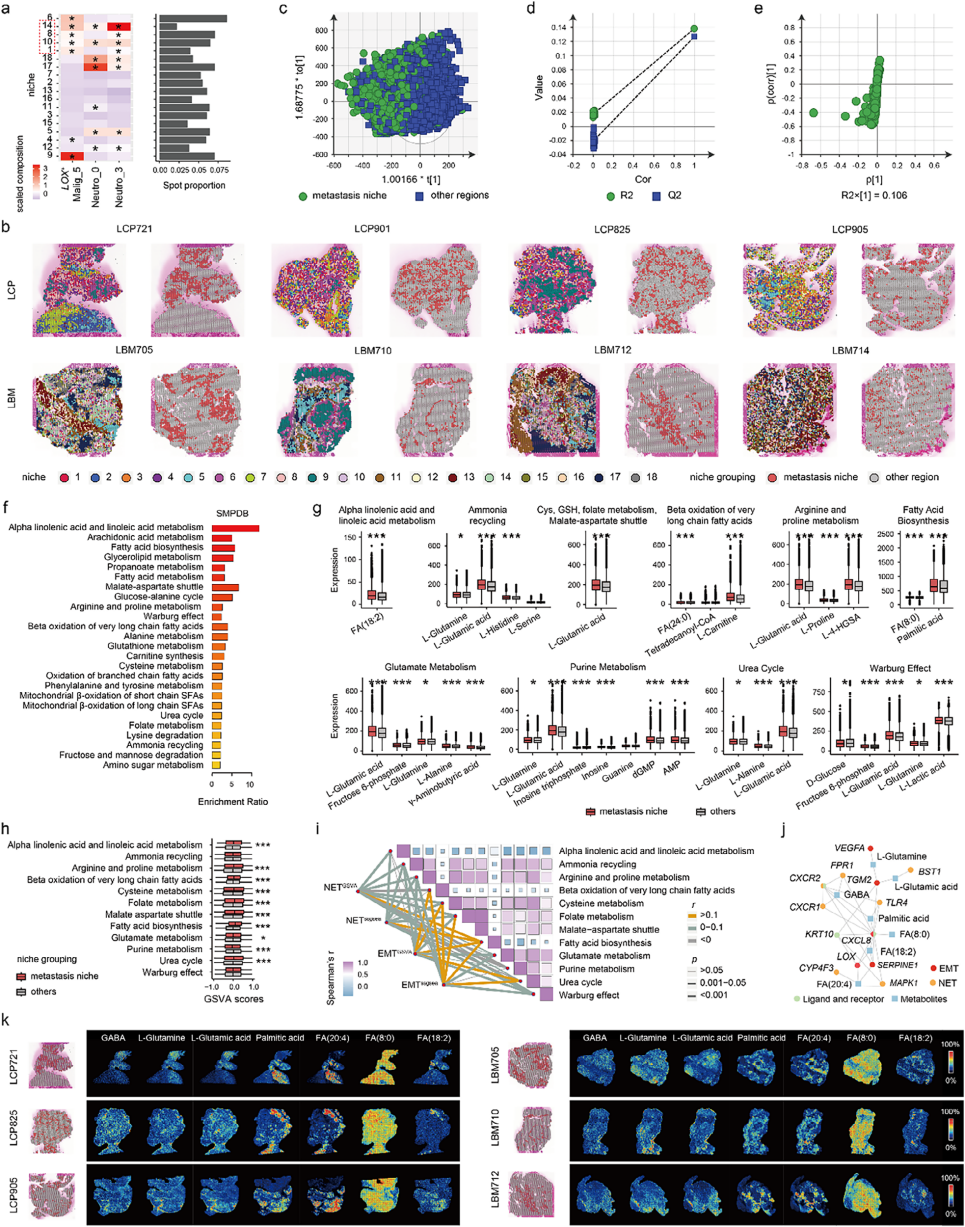
Neutro‐0, Neutro‐3, and *LOX* ^+^ Malig‐5 form a metastatic niche with metabolic reprogramming. a) Scaled median cell‐type compositions (left) and spot proportion (right) for each niche. Asterisks indicate increased composition of a cell type in a niche compared with other niches (one‐tailed Wilcoxon test, *p* < 0.05). The dashed outline highlights the niches where the composition of *LOX* ^+^ Malig‐5s and Neutro‐0s or Neutro‐3s was simultaneously increased, identifying them as the metastatic niche. b) Visualization of the spatial distribution of 18 niches and their grouping (metastatic niche and other region) on LCP and LBM spatial transcriptomic slides. c–e) OPLS‐DA analysis of spatial metabolic profiles: c) score plot, d) validation plot (permutation test), and e) S‐plot comparing metastatic niche and other regions. f) Bar plot of enriched SMPDB metabolic pathways for differential metabolites between the metastatic niche and other regions, analyzed using MetaboAnalyst. g) Comparison of metabolite expression in enriched pathways between the metastatic niche and other regions. h) Comparison of enriched pathway scores, quantified by GSVA, between the metastatic niche and other regions. Two‐tailed Wilcoxon test. * *p* < 0.05, *** *p* < 0.001. i) Spearman correlation between NET/EMT scores and enriched pathway scores. j) Gene–metabolite interaction network based on EMT/NET gene sets, metabolites in EMT/NET‐correlated pathways, and ligand–receptor pairs, constructed using MetaboAnalyst. k) Spatial distribution of metabolites in the interaction network, along with the metastatic niche region, visualized across LCP and LBM spatial metabolomics slides. AMP, adenosine monophosphate; Cys, cysteine; dGMP, 2′‐deoxyguanosine 5′‐monophosphate; FA, fatty acid; γ‐aminobutyric acid, GABA; GSH, glutathione; L‐4‐HGSA, L‐4‐hydroxyglutamate semialdehyde.

Because Neutro‐0, Neutro‐3, and *LOX* ^+^ Malig‐5 exhibited strong colocalization and interaction, we further investigated whether these cells contributed to the formation of a specific microenvironment during metastasis. First, we used the proportion of these cells to conduct niche division of spots in the spatial transcriptomic data, following previously reported methods.^[^ ^24^ ^]^ Eighteen niches were identified, with niche‐1, niche‐8, niche‐10, and niche‐14 exhibiting high expression of *LOX* ^+^ Malig‐5s, Neutro‐0s, and Neutro‐3s (**Figure** **5**a). Given the metastasis‐promoting roles of Neutro‐0s, Neutro‐3s, and *LOX* ^+^ Malig‐5s, we defined niche‐1, niche‐8, niche‐10, and niche‐14 as metastatic niches (Figure 5a,b), a concept proposed in previous studies.^[^ ^25^ ^]^

Metabolic reprogramming is a hallmark of tumor cells that supports growth, immune evasion, and metastasis. Analysis of the scRNA‐seq data revealed the unique metabolic characteristics of Neutro‐0, Neutro‐3, and *LOX* ^+^ Malig‐5 (Figure 2m; Figure S8g, Supporting Information). Herein, we compared the spatial metabolism between metastatic niches and other regions using spatial metabolomic data from paired spatial transcriptomic samples. Orthogonal partial least squares discriminant analysis (OPLS‐DA) demonstrated a distinct separation of spots between metastatic niches and other regions (Figure 5c), and 186 differentially expressed metabolites (variable importance of projection [VIP] > 0.5, *p *< 0.05, Student's *t*‐test) (Figure 5e). The permutation test indicated that the OPLS‐DA model was reliable and did not overfit (Figure 5d). Next, we imported these differentially expressed metabolites into MetaboAnalyst 6.0 for small molecule pathway database (SMPDB) metabolic pathway enrichment analysis, which identified 25 significantly enriched pathways, including alpha‐linolenic acid and linoleic acid metabolism, arachidonic acid metabolism, fatty acid biosynthesis, and fatty acid metabolism (Figure 5f). Most metabolites, such as palmitic acid, were significantly upregulated in metastatic niches compared with other regions (Figure 5g). This finding is consistent with the gene set variation analysis (GSVA) quantification analysis, which showed that the levels of most pathways, including fatty acid biosynthesis and beta oxidation of very‐long‐chain fatty acids, were also significantly increased in metastatic niches relative to other regions (Figure 5h). Among these, fatty acid biosynthesis was positively associated with both the EMT and NET levels (Figure 5i). To gain a more comprehensive understanding of the NSCLC metastasis mechanism, we utilized MetaboAnalyst 6.0 to construct a gene‐metabolite interaction network, based on EMT and NET gene sets, metabolites in EMT/NET‐correlated pathways, and ligand–receptor pairs (Figure 5j). The spatial distribution of interacting metabolites was visualized across spatial metabolomic slides (Figure 5k). Collectively, these results highlight the metabolic reprogramming within the metastatic niche formed by Neutro‐0s, Neutro‐3s, and *LOX* ^+^ Malig‐5s.

### 2.9 Palmitic Acid as a Key Metabolite of the Metastatic Niche in NSCLCs

**Figure 6:**
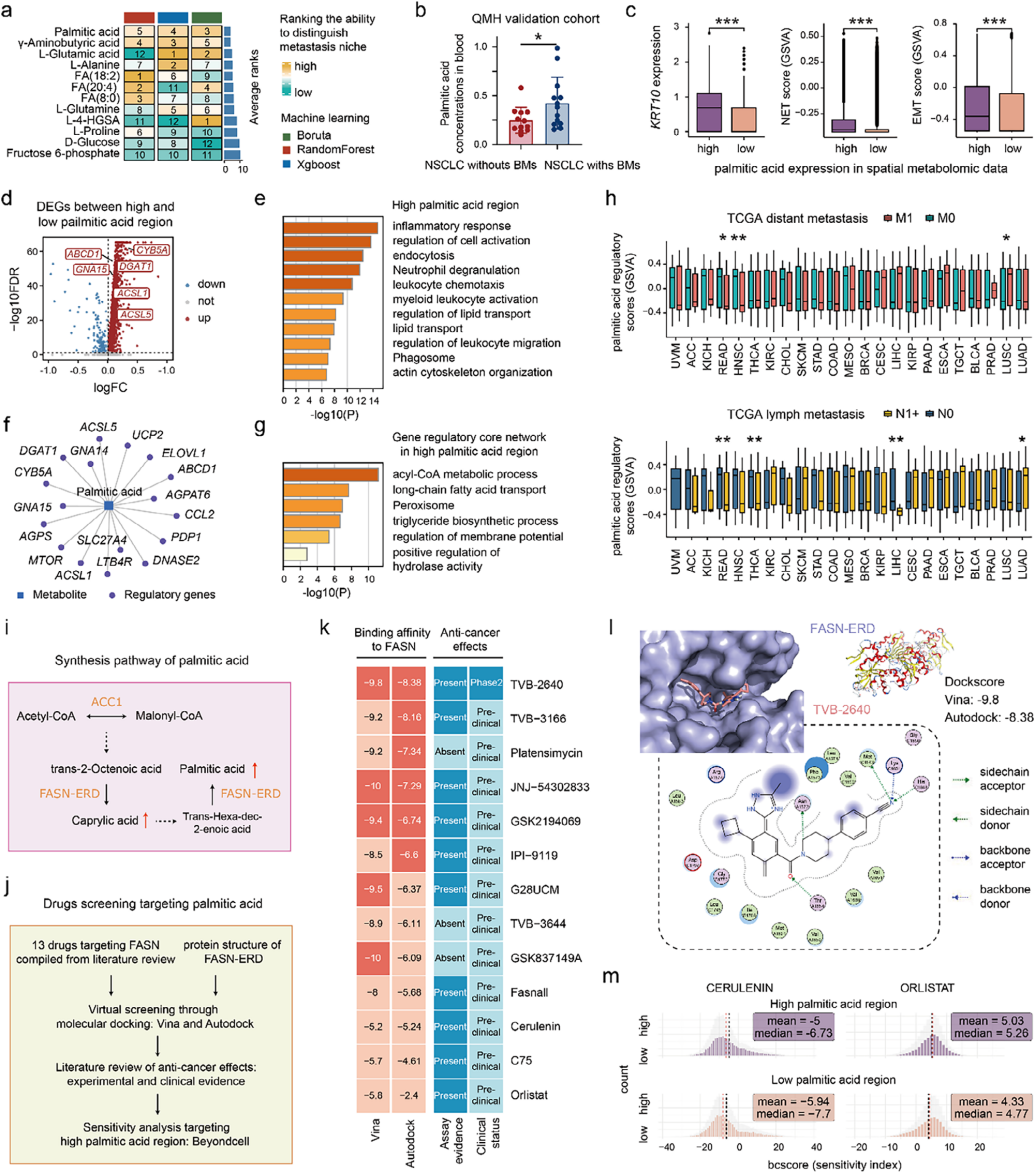
Palmitic acid is a potential key driver of the metastatic niche, and identifying therapeutic drugs that target palmitic acid to inhibit metastasis. a) Ranks of metabolites' abilities to distinguish metastatic niche and other regions using Boruta, RandomForest, and XGBoost machine learning algorithms. Palmitic acid is the most discriminatory metabolite between these two regions. b) Palmitic acid levels in the blood of patients with NSCLCs with or without BMs from the metabolic mass spectrometry data of the QMH validation cohort. c) Comparison of *KRT10* expression, NET score, and EMT scores between high and low palmitic acid regions in the LCP and LBM spatial sequencing slides. Two‐tailed Wilcoxon test. *** *p* < 0.001. d) Volcano plot showing differentially expressed genes (DEGs) between high and low palmitic acid regions. e) GO enrichment analysis of DEGs between high and low palmitic acid regions, analyzed using Metascape. f) Core regulatory network of palmitic acid constructed using DEGs from high and low palmitic acid regions, analyzed with MetaboAnalyst. g) GO enrichment analysis of palmitic acid core regulatory genes, analyzed using Metascape. h) Comparison of palmitic acid regulatory scores, quantified by GSVA using core regulatory genes, between primary cancers with and without metastasis (distant, top, or lymph node, bottom) in the TCGA pan‐cancer cohort. Two‐tailed Wilcoxon test. * *p* < 0.05, ** *p* < 0.01. i) Map of the palmitic acid synthesis process, drawn based on the SMPDB fatty acid biosynthesis pathway. The solid red arrow represents metabolites that are significantly upregulated in the metastatic niche, compared to other regions. The solid black arrow represents a direct transformation process, while the dashed black arrow represents a process where some steps are omitted. j) Workflow of virtual drug screening targeting palmitic acid. k) Identification of promising therapeutic drugs targeting palmitic acid based on FASN‐ERD binding affinity calculations (Vina and Autodock) and anti‐cancer evidence search. l) Visualization of docking pockets (top) and interactive forces (bottom) between FASN‐ERD and TVB‐2640, using PyMol and MOE, separately. m) Comparison of FASN inhibitor response assessed by Beyondcell between high and low palmitic acid regions. ACC1, Acetyl‐CoA carboxylase 1.

To identify the key metabolites driving metastatic niche formation and progression, we ranked the discriminative abilities of metabolites between metastatic niches and other regions using three machine learning algorithms: Boruta, Random Forest, and XGBoost. Palmitic acid was the most discriminatory metabolite between the two regions (**Figure** **6**a). This was supported by metabolic mass spectrometry data from the Queen Mary Hospital NSCLC (QMH‐NSCLC) cohort, which revealed significantly higher palmitic acid levels in the blood of NSCLC patients with BMs than in those without BMs (Figure 6b). Subsequently, the spatial metabolomic spots were categorized into high‐ and low‐palmitic acid regions based on the 50% median expression of palmitic acid. High‐palmitic acid regions showed significantly higher levels of KRT10, NET, EMT, *LOX* ^+^ Malig‐5, Neutro‐0s, and Neutro‐3s than the low‐palmitic acid regions (Figure 6c; Figure S12a, Supporting Information). The gene–metabolite network revealed a close interaction between palmitic acid and the EMT/NET process (Figure 5j). In vitro, palmitic acid exposure induced EMT in NSCLC cells, as suggested by the upregulation of fibronectin and downregulation of E‐cadherin (Figure S12b, Supporting Information). In addition, palmitic acid treatment increased LOX expression and promoted actin cytoskeleton remodeling, including a transition from polygonal to spindle‐like morphology, invadopodia formation, and the appearance of condensed actin foci in NSCLC cells (Figure S12c, Supporting Information). These findings indicate that palmitic acid is a key driver of the metastatic niche in NSCLCs.

Next, we investigated the source of the elevated palmitic levels in metastatic niches and their potential role in promoting metastasis. Compared with *LOX* ^+^ Malig‐5 in the malignant cell subclusters (ranking 12/17), Neutro‐3 and Neutro‐0 exhibited higher activity for long‐chain saturated fatty acid biosynthesis (ranking 4/11 and 6/11, respectively) (Figure S13, Supporting Information), indicating that neutrophils, and not malignant cells, are probably the main source of palmitic acid in metastatic niches, with diet as another possible contributor. Moreover, owing to the lack of metabolic data on pan‐cancer metastasis, we used the core regulatory network of palmitic acid to investigate its potential role in driving metastasis across cancers. Based on the 4106 upregulated genes (log2FC > 0, *p* < 0.05) identified in the high palmitic acid regions (Figure 6d,e), we constructed a core palmitic acid regulatory network using MetaboAnalyst 6.0, containing 17 regulatory genes (Figure 6f). This network showed functional enrichment for long‐chain fatty acid transport and triglyceride biosynthesis (Figure 6g). Palmitic acid regulatory scores were calculated in the TCGA pan‐cancer cohort using GSVA. Only lung adenocarcinoma and lung squamous cell carcinoma exhibited significantly higher palmitic acid regulatory scores in tumors with distant or lymphatic metastasis, in comparison to those without (Figure 6h), further confirming the metastasis‐promoting of palmitic acid in NSCLCs. Although palmitic acid promotes metastasis in multiple cancers,^[^ ^26^, ^27^ ^]^ its mechanism of action in NSCLCs may be unique.

### 2.10 Identifying Therapeutic Drugs That Target Palmitic Acid to Inhibit Metastasis

Palmitic acid may play a crucial role in metastatic niches. Palmitic acid, the end product of fatty acid biosynthesis, is primarily synthesized via the action of the fatty acid synthase enoyl reductase domain (FASN‐ERD) (Figure 6i). Previous studies have identified fatty acid synthase (FASN) as a classical oncology target and developed FASN‐targeting pharmacological agents.^[^ ^28^ ^]^ However, the most effective drugs remain unclear. Therefore, we systematically selected 13 FASN inhibitors from previous studies^[^ ^28^, ^29^ ^]^ and conducted virtual drug screening, including molecular docking and sensitivity analyses (Figure 6j). Molecular docking analysis revealed that TVB‐2640 had the lowest binding score for FASN‐ERD, indicating the highest affinity and potential for inhibiting FASN‐ERD function (Figure 6k). Both experimental and clinical (phase 1/2 trials) evidence^[^ ^30^, ^31^, ^32^ ^]^ has reported the anticancer effects of TVB‐2640, whereas all other FASN inhibitors remain in preclinical research (Figure 6k). The docking pockets and interactive forces between TVB‐2640 and FASN‐ERD were visualized using PyMol and MOE (Figure 6l). In addition, since there are currently no available drug sensitivity data for TVB‐2640, we used the other FASN inhibitors, Cerulenin and Orlistat, to perform beyond‐cell drug sensitivity analysis in the spatial data of NSCLCs. Regions with high palmitic acid levels were more sensitive to Cerulenin and Orlistat than were regions with low palmitic acid levels (Figure 6m), indicating the inhibitory effects of FASN inhibitors on NSCLC metastasis. In laboratory studies, TVB‐2640 treatment reduced LOX expression and actin cytoskeleton remodeling (encompassing polygonal‐to‐spindle morphology transformation, invadopodia formation, and condensed spot emergence) in NSCLC cells, whereas palmitic acid exposure reversed these suppressive effects (Figure S12d, Supporting Information). Similar results were observed in the tail vein injection metastasis model, where TVB‐2640 suppressed NSCLC growth and prolonged animal survival, while palmitic acid enhanced NSCLC metastasis (Figure S14a–d, Supporting Information). Overall, our findings identified FASN inhibitors, such as TVB‐2640, as potential treatments for NSCLC BMs by inhibiting palmitic acid synthesis.

## 3 Discussion

**Figure 7:**
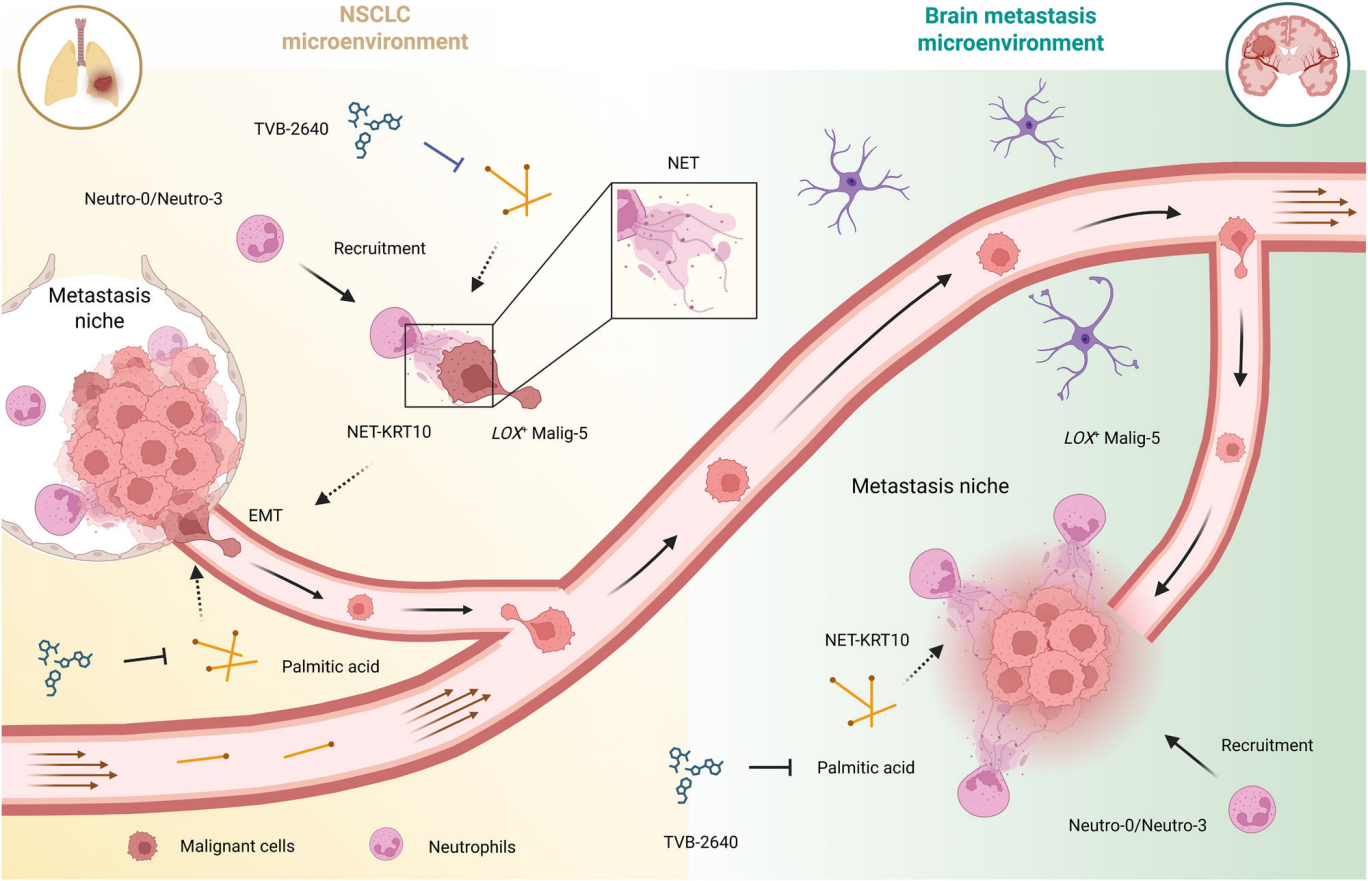
Summary of this study. A *LOX* ^+^‐expressing malignant cell subcluster (Malig‐5) is MICs during NSCLC BMs. These cells contribute to the formation of metastatic niches alongside neutrophil subtypes (Neutro‐0 and Neutro‐3) that are predisposed to releasing NETs. The NET–KRT10 signaling axis mediates the interaction between NET‐releasing neutrophils and *LOX* ^+^‐NSCLC cells, thereby promoting EMT and metastatic dissemination. The elevation of palmitic acid levels in the resulting metastatic niche emerges as a crucial metabolic driver of BMs. Inhibition of palmitic acid using FASN inhibitors (e.g., TVB‐2640) can become a potential therapeutic approach to disrupt this metabolic vulnerability and suppress NSCLC BMs. Created with BioRender.com.

Metabolic reprogramming has been implicated in driving tumor metastasis; however, traditional omics techniques remain limited in capturing metabolic heterogeneity within tumors and fail to fully elucidate the complex metabolic interactions between different cells in the metastatic niche. In this study, we utilized spatial, single‐cell, and bulk multi‐omics sequencing technologies to explore cellular interactions and metabolic heterogeneity within NSCLC BMs. *LOX* ^+^ Malig‐5 represented the MICs and formed a metastatic niche in conjunction with NET‐releasing neutrophil subtypes, interacting mainly through the NET–KRT10 axis. The metastatic niche is involved in metabolic reprogramming, with palmitic acid serving as the key metabolite. FASN inhibitors may be promising therapeutic agents that target palmitic acid to inhibit the BM in NSCLCs (**Figure** **7**).

MICs are a rare subset of cancer cells with the unique ability to initiate and sustain metastasis.^[^ ^4^ ^]^ Several biomarkers have been identified that mark MICs, including CD44,^[^ ^33^ ^]^ CXCR3,^[^ ^34^ ^]^ CD36,^[^ ^6^ ^]^ LMO2,^[^ ^35^ ^]^ and PDX1^[^ ^36^ ^]^ in various tumors. In this study, we screened *LOX* ^+^ Malig‐5 as the MICs for NSCLC BMs through comprehensive analyses, including pseudotime, deconvolution, and functional enrichment. *LOX* ^+^ Malig‐5 cells exhibited high stemness and hybrid EMT status, consistent with previously reported features of MICs.^[^ ^37^ ^]^ LOX, a cell marker of Malig‐5, is a copper‐dependent enzyme that oxidizes lysine residues in collagen and elastin, promoting extracellular matrix remodeling through covalent cross‐linking.^[^ ^38^ ^]^ LOX facilitates breast cancer metastasis to the lungs and bones by remodeling the extracellular matrix within the metastatic niche.^[^ ^39^, ^40^ ^]^ Additionally, LOX induces EMT in hepatocellular carcinoma metastasis,^[^ ^41^ ^]^ which is consistent with our findings.

Neutrophils are key players in the metastatic microenvironment, involved in each stage of metastasis, and exhibit heterogeneity in multiple cancers.^[^ ^42^, ^43^ ^]^ Previous studies have classified neutrophils into immature and mature,^[^ ^12^ ^]^ as well as N1 (antitumor) and N2 (protumor).^[^ ^44^ ^]^ In our study, we observed strong spatial colocalization between *LOX* ^+^ Malig‐5s and NET‐releasing neutrophils, which could predict NSCLC metastasis. NETs are web‐like extracellular structures released by activated neutrophils.^[^ ^45^ ^]^ Sprenkeler et al. have reported that NET formation depends on cytoskeletal rearrangements in neutrophils.^[^ ^46^ ^]^ Interestingly, NETs bind to tumor cells via keratin, a cytoskeletal component. Specifically, pull‐down assays and mass spectrometry demonstrated a strong NET‐binding capacity of *LOX* ^+^ Malig‐5s, with KRT10 emerging as the pivotal tumor protein that mediates *LOX* ^+^ Malig‐5 and NET interactions. KRT10 (Keratin 10) is a type I intermediate filament protein that provides structural support to epithelial cells.^[^ ^47^ ^]^ Our study revealed a positive correlation between *KRT10* expression and EMT scores in NSCLC cells, which is consistent with the report by Zhang et al. on KRT10 upregulation during EMT.^[^ ^48^ ^]^ Moreover, KRT10 knockdown significantly reduced NSCLC cell invasion and metastasis in the NET microenvironment, consistent with previous findings in laryngeal cancer.^[^ ^47^ ^]^ Based on these results, we propose that neutrophil‐mediated metastasis occurs through NETs–KRT10 binding, which promotes EMT in *LOX* ^+^ Malig‐5 cells, underscoring the immunoregulatory function.^[^ ^49^ ^]^

Interacting cells are often highly colocalized, enabling them to form specialized microenvironments, known as niches.^[^ ^50^ ^]^ In this study, we identified the metastatic niche formed by MICs and their interacting NET‐releasing neutrophils using niche clustering analysis. Metabolic reprogramming of metastatic niches has been reported, including neutral lipid accumulation, glucose enrichment, and oxalate increase, all of which can facilitate tumor cell metastasis.^[^ ^51^, ^52^, ^53^ ^]^ In our study, spatial metabolomic analysis revealed metabolic reprogramming within the metastatic niche, with a particular focus on palmitic acid upregulation. Palmitic acid, one of the most abundant saturated fatty acids in the human body, plays a crucial role in cell membrane construction, fat metabolism, and energy production, as it promotes tumor cell growth and metastasis.^[^ ^54^ ^]^ Pascual et al. have reported that dietary palmitic acid induces long‐lasting prometastatic memory in cancer cells through Schwann cell activation.^[^ ^26^ ^]^ Manzano et al. have reported that a palmitate‐rich environment enhanced metastatic growth through p65 acetylation.^[^ ^55^ ^]^ Our study further showed that high palmitic acid regions had elevated levels of NET and EMT, with the gene–metabolite network revealing a close interaction between palmitic acid and NET/EMT, which is consistent with previous findings on the role of palmitic acid in inducing EMT in epithelial cells and NET release in neutrophils.^[^ ^56^, ^57^ ^]^ Palmitic acid levels were elevated not only in the metastatic niche but also in the blood of patients with NSCLC with BMs, implying its potential as a metastasis biomarker. Supporting this, fatty acid metabolism‐related metabolites and enzymes are increasingly being recognized as biomarkers for cancer prediction and prognosis.^[^ ^58^ ^]^ Moreover, Rayes et al. observed higher circulating NET levels in patients with advanced versus localized lung cancer,^[^ ^59^ ^]^ indicating that palmitic acid may induce NET formation in both the circulation and metastatic niches.

Next, we investigated the source of elevated palmitic acid levels in the NSCLC BM and found that palmitic acid might be generated from exogenous dietary and/or metastatic niches. We recommend limiting the intake of palmitic acid‐rich foods (e.g., palm oil, butter, and fatty meat) for NSCLC patients. Palmitic acid biosynthesis was regulated in the metastatic niche; therefore, we aimed to target FASN‐ERD, a key enzyme involved in palmitic acid biosynthesis, as a strategy to suppress NSCLC BMs. FASN has been previously identified as a classical oncology target.^[^ ^28^ ^]^ Here, we analyzed 13 FASN inhibitors and observed that TVB‐2640 had the greatest potential to inhibit FASN‐ERD function. The anticancer effects of TVB‐2640 have been demonstrated in previous experimental assays and clinical trials involving multiple tumors, including NSCLCs, astrocytoma, ovarian cancer, breast cancer, and others.^[^ ^31^, ^32^, ^60^ ^]^ The efficacy of TVB‐2640 in patients with astrocytoma indicates its potential blood–brain barrier penetration.^[^ ^31^ ^]^ In addition, TVB‐2640 treatment reduced LOX expression and actin cytoskeleton remodeling in NSCLC cells, whereas palmitic acid exposure reversed these suppressive effects, supporting TVB‐2640′s role in blocking NSCLC metastasis. Cerulenin and Orlistat, the other two FASN inhibitors, exhibited higher sensitivity to high palmitic acid regions, indicating the potential inhibitory effects of FASN inhibitors on NSCLC BMs. These findings are similar to those of previous studies reporting that FASN inhibitors can effectively inhibit palmitic acid‐driven metastasis of breast and ovarian cancers to the lungs.^[^ ^60^ ^]^ Collectively, our findings demonstrate the potential of FASN inhibitors for the prevention and treatment of NSCLC, underscoring the urgent need for further experimental and clinical studies.

However, this study had some limitations. First, the relatively small size of spatial multi‐omics cohorts may limit the statistical power and generalizability. Future studies should include larger and more diverse populations to validate our conclusions. Second, we observed a limited EMT marker response in BM101 and A549 cells upon palmitic acid exposure compared to PC9 cells. These results may reflect cell line‐specific differences in the basal EMT status, with BM101 and A549 being mesenchymal‐like and PC9 being epithelial‐like. Third, in the A549 tail vein injection model, metastases were found only in the thoracic and abdominal cavities after lung colonization, with no clear evidence of brain metastasis. As A549 cells lack inherent brain metastatic potential, they require longer times and show low brain colonization efficiency. Future studies using BM101‐based models are needed to better assess brain metastasis.

In conclusion, our study provides a high‐resolution spatial cellular and molecular atlas of NSCLC BM. Through spatial, single‐cell, and bulk multi‐omics analyses, we identified *LOX* ^+^ Malig‐5 as the MICs for NSCLC BMs. MIC *LOX* ^+^ Malig‐5 and NET‐released neutrophils collaborated to form a metastatic niche with metabolic reprogramming. Additionally, FASN inhibitor TVB‐2640 targeting palmitic acid could be a promising therapeutic strategy for NSCLC BMs.

## 4 Experimental Section

### Ethical Approval and Informed Consent

This multicenter study was approved by the Medical Ethics Committee of Xiangya Hospital, Central South University (No. 2 022 020 484) and the Institutional Review Board (IRB) of the University of Hong Kong/Hospital Authority Hong Kong West Cluster (HKU/HA HKWIRB, IRB Reference Number: UW07‐273). All patients provided written informed consent for sample collection and data analysis before surgery in accordance with the Declaration of Helsinki and Good Clinical Practice guidelines. All animal experiments were performed in compliance with the animal research: reporting of in vivo experiments (ARRIVE) guidelines and the regulations of the Animal Welfare Committee of Xiangya Hospital (Animal ethics approval number: 2 023 030 607).

### Patient Cohorts and Sample Collection

Nine patients with NSCLC who underwent surgical resection of primary tumors, adjacent lymph nodes, and BMs at Xiangya Hospital were enrolled, and matched tumor and blood biospecimens were collected. Spatial multiomics (transcriptomics and metabolomics) sequencing was performed on four primary tumors and four BMs, whereas paired single‐cell RNA sequencing (scRNA‐seq) was conducted on five primary tumors and four BMs. Moreover, previously published in‐house blood metabolomic data were supplemented from 25 patients with NSCLC (with/without BMs) at Queen Mary Hospital, which was published previously.^[^ ^61^ ^]^ External scRNA‐seq data from public NSCLC cohorts were obtained from the Gene Expression Omnibus (https://www.ncbi.nlm.nih.gov/geo/, GSE131907), whereas bulk RNA sequencing (RNA‐seq) data from NSCLC and pan‐cancer cohorts were sourced from the Cancer Genome Atlas (TCGA, https://portal.gdc.cancer.gov/).

### BD Rhapsody scRNA‐Seq and Data Pre‐Processing

According to 10× Genomics recommended experimental procedures (CG000147), freshly collected tumor tissues were cut into 2–4 mm^3^ segments and lysed using mechanical dissociation with a gentleMACS Octo Dissociator (Cat No. 130‐096‐427, Miltenyi Biotec, USA) and enzymatic digestion with lyophilized enzymes A, B, and D. The lysate was filtered and centrifuged, red blood cells were removed, and the cell number and viability were determined to produce a single‐cell suspension. Subsequently, scRNA‐seq was performed using the BD Rhapsody platform (BD Biosciences, U.S.A.). Following the manufacturer's recommendations, the single‐cell suspension was adjusted to the appropriate volume for loading and capture using the BD Rhapsody Enhanced Cartridge Reagent Kit (Cat. No. 664 887) and BD Rhapsody Cartridge Kit (Cat No. 633 733). Reverse transcription was performed using the BD Rhapsody cDNA kit (Cat No. 633 773). BD Rhapsody WTA amplification kit (Cat No. 633 801) was used for DNA library construction. High‐throughput sequencing was performed in PE‐150 mode.

Raw FASTQ data were processed using the BD WTA rhapsody analysis pipeline (https://bitbucket.org/CRSwDev/cwl/src/master/) to generate cell‐by‐gene count matrices, which were subsequently imported into the R package “Seurat” (v 5.0.1). Low‐quality cells were excluded based on the criteria nFeature_RNA > 100 and percentage mt < 20. Count matrices were normalized and scaled using the “NormalizeData” and “ScaleData” functions, and their dimensionality was reduced by principal component analysis on the top 2000 highly variable genes. Subsequently, the batch effect between different tumor samples was removed using the R package “harmony” (v 1.1.0), followed by further dimensional reduction of uniform manifold approximation and projection (UMAP). Cells were clustered by the “FindNeighbors” and “FindClusters” functions with the default settings. Cell types of each cluster were automatically annotated using the R package “scHCL” (v 0.1.1), and then manually corrected based on the expression of canonical markers, which were collected from previous studies.^[^ ^62^, ^63^, ^64^ ^]^ Specifically, canonical markers include B cells (*CD79A* and *IGHG3*), dendritic cells (DCs) (*IL3RA* and *IRF7*), endothelial cells (*VMF* and *PECAM*), epithelial cells (*CDH1* and *EPCAM*), fibroblasts (*THY1* and *DCN*), macrophages (*MRC1* and *CD163*), mast cells (*MS4A2* and *KIT*), neuroglial cells (*OLIG1* and *OLIG2*), neutrophils (*S100A8* and *S100A9*), natural killer (NK) cells (*GNLY* and *NKG7*), and T cells (*CD3E* and *CD3D*).

### 10× Visium CytAssist Spatial Transcriptomics Sequencing and Data Pre‐Processing

Freshly collected tumor tissues were divided into appropriate sizes (6.5 mm × 6.5 mm) and embedded in optimal cutting temperature compound and quickly frozen on dry ice. Tissue sections were subjected to methanol fixation, hematoxylin–eosin staining, imaging, and destaining following the 10× Genomics recommended experimental procedures (CG000614). According to the 10× Genomics experimental flow (CG000495), probe hybridization, probe release, and transfer to the 10× Visium CytAssist slide, and the library construction was performed using the Visium CytAssist spatial gene expression for FFPE kit (Cat No. PN‐1 000 520, 10× Genomics, U.S.A.). The DNA libraries were subjected to high‐throughput sequencing in the PE‐150 mode. Raw FASTQ data were quality controlled and aligned to the reference genome GRCh38 using Space Ranger (v 3.1.1) and STAR (v 2.7.11b) to produce spot‐by‐gene count matrices. Next, normalization across spots was conducted using the “SCTransform” function of the R package “Seurat” (v 5.0.1). Matrix dimension was reduced by the “RunPCA” function, and the batch effect between different slides was corrected through the R package “harmony” (v 1.1.0).

### Desorption Electrospray Ionization Mass Spectrometry Imaging (DESI‐MSI) and Spatial Metabolomics Data Pre‐Processing

The embedded tumor samples were stored at −80 °C before being sectioned. The samples were cut into 10 consecutive sagittal slices, each ≈10 µm thick using a cryostat microtome (Leica CM 1950, Leica Microsystem, Germany) and were thaw‐mounted on positive charge desorption plates (Thermo Scientific, USA). Sections were stored at −80 °C before further analysis. They were desiccated at −20 °C for 1 h and then at room temperature for 2 h before mass spectrometry imaging (MSI) analysis. An adjacent slice was stained with H&E.

The analyses were performed as previously reported.^[^ ^65^ ^]^ Briefly, this experiment was conducted using an AFADESI‐MSI platform (Beijing Victor Technology Co., LTD, China) in tandem with a Q‐Orbitrap mass spectrometer (Q Exactive, Thermo Scientific, USA). The solvent formula was acetonitrile (ACN)/H_2_O (8:2) at negative mode and ACN/H2O (8:2, 0.1%FA) at positive mode and the solvent flow rate was 5 µL min^−1^, the transporting gas flow rate was 45 L min^−1^, the spray voltage was set at 7 kV, and the distance between the sample surface and sprayer was 3 mm as was the distance from the sprayer to the ion transporting tube. The mass spectrometry resolution was set at 70 000, the mass range was 70–1000 Da, the automated gain control target was 2 × 10^6^, the maximum injection time was set to 200 ms, the S‐lens voltage was 55 V, and the capillary temperature was 350 °C. The MSI experiment was performed with a constant rate of 0.2 mm s^−1^, continuously scanning the surface of the sample section in the x direction and a 50 µm vertical step in the y direction. Next, the collected raw files were converted into imML format using imzML converter and then imported into MSiReader (an open‐source interface to view and analyze high‐resolution power mass spectrometry‌ imaging files on the MATLAB platform) for ion image reconstructions after background subtraction using the Cardinal software package. All mass spectrometry images were normalized using total ion count normalization for each pixel.

### Cellular Pseudotime Trajectory and RNA Velocity Analyses

To predict the cellular differentiation states of malignant cells and neutrophils, the R package “CytoTRACE” (v 0.3.3) with the default parameter was employed. Higher CytoTRACE scores signified lower differentiation and vice versa.^[^ ^66^ ^]^ Next, the R package “Monocle” (v 2.30.0) was utilized to characterize the cellular developmental pseudotime trajectory, with the origins identified from CytoTRACE results. Highly variable genes along pseudoprime were determined using the “DifferentialGeneTest” function, followed by GO enrichment analysis (*p* < 0.05). The constructed pseudotime trajectories were validated using other pseudotime algorithms, including diffusion map (R package “destiny,” v 3.16.0) and pyvia (python package “pyvia,” v 0.1.77). The first component was extracted for the diffusion map, and the coefficients of this component were displayed.^[^ ^67^ ^]^ For Pyvia, the default parameter settings were used, and differentiation origins were identified based on the CytoTRACE results. Subsequently, pseudotime agreement was assessed by calculating the two‐way random intraclass correlation coefficient across the CytoTRACE, Monocle2, diffusion map, and PyVIA results. To further validate these trajectories, the R package “velocyto.R” (version 1.16.0) was employed to calculate RNA velocity, based on the count matrices for spliced and unspliced reads extracted from loom files. The velocity vector fields were visualized using the function “show.velocity.on.embedding.cor” to assess the direction of cellular differentiation.

### Cell Type Deconvolution in Bulk RNA‐Seq, and Clinical Metastasis and Prognosis Analyses

To assess which subtype abundance of malignant cells and neutrophils could serve as a predictor of NSCLC metastasis, the R package “BayesPrism” (v 2.1.2) was employed to estimate the fractions of cell type in bulk RNA‐seq data of TCGA‐NSCLC tumors, with the scRNA‐seq data of the in‐house primary NSCLC tumors as reference. Cell‐type annotation was used, except for malignant cells and neutrophils, for which both cell types and subtypes were considered. Moreover, according to the tutorial, ribosomal protein genes, mitochondrial genes, and genes from chromosomes M, X, and Y were excluded, and only protein‐coding genes were considered, to mitigate batch effects and enhance computational efficiency. Subsequently, the proportion of each subpopulation of malignant cells and neutrophils was compared between tumors with and without metastasis in patients with TCGA‐NSCLC. An AUC analysis was also conducted to compare the ability of each subpopulation to predict metastasis. Metastasis status was categorized according to TMN staging (N0, no lymph node metastasis; N1–3, lymph node metastasis; M0, no distant metastasis; M1, distant metastasis). In addition, patients with TCGA‐NSCLC were stratified into two groups (high and low) using the median of each subpopulation proportion as a cutoff. Survival data were analyzed, Kaplan–Meier survival curves were generated, and a log‐rank test was used to evaluate the differences between groups. An AUC analysis was also performed to compare the predictive ability of each subpopulation in determining patients with NSCLCs.

### Cell Type Deconvolution in Spatial Transcriptomic Data

To spatially locate the cell types on the spatial transcriptomics slides, the Python package “Cell2location” (v 0.9.0) was utilized. scRNA‐seq data from paired patients with NSCLC were used as references. Cell type annotation was used, except for malignant cells and neutrophils, for which both cell type and subtype were considered. Moreover, according to the tutorial, mitochondrial genes and lowly expressed genes (those expressed in fewer than five cells or with a mean expression lower than log_10_2) were excluded. Spatial deconvolution was conducted using the “cell2location.models.Cell2location” and “mod.train” functions. The default parameters were used, except for N_cells_per_location, which was set to 30, and max_epochs, which was set to 3000. Each spatial transcriptomic section was analyzed separately. The results were visualized using the Cell2location tutorial, with plots depicting the estimated abundance of cell types or cell subtypes.

### Spatial Colocalization Analyses

The R package “mistyR” (v 1.10.0) was used to dissect the spatial colocalization relationships among each cell type and subtype, based on the Cell2location deconvolution matrix. Cell type annotation was used, except for malignant cells and neutrophils, for which both cell types and subtypes were considered. The analysis was conducted following the “mistyR” tutorial, using a multi‐view model: 1) intrinsic view, which measured the relationships within a spot, 2) juxta view, which evaluated the relationships of adjacent spots, 3) para view, which characterized the relationships of more distant neighbors (effective radius = three spots). The colocalization results across all slides were aggregated for each view and were visualized through the “plot_interaction_heatmap” and “plot_interaction_communities” functions.

### Multiplex Immunofluorescence Staining Assay

For multiplex immunofluorescence staining, the paraffin sections were boiled in antigen unmasking solution, blocked with 5% bovine serum albumin for 10 min, and treated for 30 min at room temperature using the following primary antibodies: CK‐19 (rabbit 1:500, GeneTex, #HL2878, China), CD31 (mouse 1:500, Cell Signaling, #3528, USA), H3Cit (rabbit, 1:500, GeneTex, #GTX122148), and MPO (Myeloperoxidase, Rabbit, 1:500, GeneTex, #GTX135126, China). The paraffin sections were rinsed three times with phosphate‐buffered saline (PBS) and incubated with horseradish peroxidase‐conjugated secondary antibodies for 10 min at room temperature. After washing three times in PBS, the sections were incubated with fluorochromes for 10 min at room temperature. Specimens were rinsed three times with PBS and then treated with 10 mm sodium citrate in the microwave at 100 °C for 3 min, and naturally cooled down to room temperature. Next, another round of blocking and a new set of primary antibody staining were performed for marker detection. This process was repeated successively to stain all three or four markers in one sample slice. 4′,6‐Diamidino‐2‐phenylindole (1:10 000, Beyotime, #C1002, China) was used for 1 min at room temperature before multiple fluorescence scanning.

### Patients’ Neutrophil Generation and Cell Culture

Neutrophils of patients coded “LBM620,” “LBM710,” “LBM705,” “LCP721,” “LCP901,” and “LCP905” from the XYNS cohort, who were independently diagnosed and confirmed by pathologists. Neutrophils were isolated from the whole blood of the patients using the Percoll Gradient (Sigma‐Aldrich, #P4937, USA). Briefly, leukocytes were collected after centrifugation and rupture of red blood cells. The following were prepared: 10× saline (NaCl: 90 g L^−1^), diluted 9‐parts Percoll in 1‐part 10× saline to make “100% Percoll” (900 mL Percoll + 100 mL 10× Saline), prepared 72% Percoll using red Hank's balanced salt solution, prepared 36% Percoll using Dulbecco's phosphate‐buffered saline. Leukocytes were resuspended in 3 mL 72% Percoll, and 3 mL 26% Percoll was added to the leukocyte suspension. The cells were centrifuged at 700 *g* for 20 min at room temperature, with no brake. The neutrophils were flattened at the liquidus interface. Washing and collection were performed as described previously.

The human NSCLC cell lines A549 (RRID: CVCL_0023) and PC9 (CVCL_S750) were purchased from the American Type Culture Collection and cultured in Gibco Dulbecco's modified Eagle medium F12 medium (Thermo, # 12 634 010, USA) with 10% fetal bovine serum (Gibco, #A5669402, USA) in a humidified 5% CO_2_ incubator at 37 °C. NSCLC patient‐derived tumor cells coded “BM101” and “BM104”^[^ ^68^ ^]^ were maintained in Neurocult NSA basal medium and proliferation supplement (Stemcell Technologies #0 5751, CAN), with 20 ng mL^−1^ EGF (Life Technologies, # PHG0314, USA), and basic 20 ng mL^−1^ FGF (Peprotech, #100‐19, USA). All the cells were free from mycoplasma contamination.

### NET DNA Isolation, Biotin‐Label, Pull‐Down Assay, and Protein Identification

Neutrophils were allowed to settle in 6‐well plates (5 × 10^5^ well^−1^) for 30 min and then stimulated with 0.2 nm phorbol 12‐myristate 13‐acetate (PMA; Sigma‐Aldrich, #p8129‐5MG, USA) for 4 h. After PMA stimulation, the cells were collected and treated with MNase (final Con. 1 U/5 µL, Sigma‐Aldrich, #N3775, USA) according to protocol; then processed DNA isolation according to BeyoMag blood genomic DNA isolation kit with magnetic beads (Beyotime, #D0091S) manufacturer's specifications. The instructions of the Biotin DNA Labeling Kit (#D3106; Beyotime, China) were followed to label neutrophil NET DNA.

A549, BM101, and BM104 tumor cells and cytosolic and membrane proteins were extracted using the Membrane and Cytosolic Protein Extraction Kit (Beyotime, #P0033, China) according to the manufacturer's instructions.

Biotin‐labeled NET DNA was pulled down with A549, BM101, and BM104 cell membrane or cytosol proteins according to the following system: 100 µL of the cell membrane or cytosol protein, 40 µL of biotin‐labeled NET DNA, 60 µL of BeyoMag Anti‐HA Magnetic Beads, 400 µL of IP lysis (Beyotime, #P0013, China) at 4 °C overnight. Beads, cell membranes, or cytosolic proteins were used as negative controls for the pull‐down assay. BM101 and BM104 membrane or cytosolic proteins were combined for pull‐down with biotin‐labeled NET DNA.

Pull‐down products were electrophoresed on an 8% sodium dodecyl sulfate–polyacrylamide gel electrophoresis and labelled using a Protein Silver Stain Kit (CWBIO, #CW2012S, China). Significant bands were cut off for proteomics mass spectrometry‌ sequencing (BGI, China). The top NET DNA pull‐down proteins in NSCLC cells were identified based on emPAI and proteomic mass spectrometry scores.

### Single‐Cell and Spatial Ligand–Receptor Interaction Analysis

Ligand–receptor interaction analysis was conducted using both scRNA‐seq and spatial transcriptomics data. For the scRNA‐seq data, the R package “CellChat” (v 1.6.1) was employed. After labeling the cell type or subtype and identifying overexpressed genes, the intercellular communication probability was computed using the “computeCommunProb” function, and the communication at the signaling pathway level was inferred by the “computeCommunProbPathway” function. Because NET DNA pulls down KRT10 in NSCLC cells, the NET–KRT10 pair was added between neutrophils and NSCLC cells to the default ligand–receptor database. Graphs were generated using the R packages “circlize” (v 0.4.15) and “ggplot2” (v 3.4.4). For the spatial transcriptomics data, the Python packages “stLearn” (v 0.4.9) and “COMMOT” (v 0.03) were utilized. Genes with low expression (those expressing fewer than five spots) were excluded, the count matrix was normalized, and highly variable genes (those with dispersion > 0.5) were screened. Next, cell–cell interaction was estimated using the “stlearn.tl.cci.run” and “commot.pl.plot_cell_communication” functions, with default parameters. The ligand–receptor scores and signal flow of the NET–KRT10 and other identified ligand–receptor pairs were visualized in each slide using the “stlearn.pl.lr_result_plot” and “commot.pl.plot_cell_communication” functions.

### Definitions of Niche and Identification of Metastatic Niche

To identify groups of spots with similar compositions of Malig‐5, Neutro‐0, and Neutro‐3 across slides, the subtype proportions of malignant cells and neutrophils were transformed into isometric log ratios using the “ilrBase” function of the R package “compositions” (v 2.0.8), and clustered the spots into different groups. These groups of spots were defined as niches, which were consistent with previous studies.^[^ ^24^ ^]^ Louvain clustering was performed by constructing a shared nearest community graph with *k* = 50, using the “buildSNNGraph” function of the R package “scran” (v 1.30.0). Statistically overrepresented cell subtypes in each niche were identified using Wilcoxon tests (false discovery rate < 0.05). Because Malig‐5 and Neutro‐0/Neutro‐3 exhibit metastasis‐initiating features and high colocalization, niches with simultaneously high proportions (scaled median cell‐subtype proportion larger than 0) of both Malig‐5 and Neutro‐0/Neutro‐3 were referred to as metastatic niches.

### Point‐To‐Point Matching for Spatial Transcriptomic and Metabolomic Data

Point‐to‐point matching between the spatial transcriptomic and metabolomic data was conducted using a patented method (CN202211278620.2). Briefly, both the barcode spatial information of the spatial transcriptome and pixel point information of the spatial metabolome were transformed into unified spatial information identifiers. The transformed pixel points of the spatial metabolome were connected to spots from the spatial transcriptome by performing a summation operation on the ion data of each pixel point corresponding to each spot. Finally, reintegrated spatial metabolomic data matching the spatial transcriptome were acquired.

### Differential Metabolite and Pathway Enrichment Analyses of Metastatic Niche

Spatial mass spectrometry profiles of the metastatic niche were precisely extracted using point‐to‐point matching between spatial transcriptomic and metabolomic data. Metabolites discriminating between the metastatic niche and other regions were screened using a supervised statistical analytical method called OPLS‐DA. The VIP values obtained from the OPLS‐DA model were used to rank the contribution of each variable to distinguish the metastatic niche from other regions. Two‐tailed Student's *t*‐test was used to verify whether the differences in metabolite levels between the two groups were significant. Next, differential metabolites were screened with VIP values > 0.5 and *p* < 0.05. The fold change in differential metabolites between the two groups was computed as *logFC*  =  *log*_2_ *metastasis* *niche*/*other* *region*. These metabolites were then imported into MetaboAnalyst 6.0 (https://www.metaboanalyst.ca/MetaboAnalyst/ModuleView.xhtml), referenced with SMPDB for enrichment analysis. The level of the enriched pathway was quantified using the GSVA method of the R package “GSVA” (v 1.50.5). The spatial distribution correlation between the EMT/NET and metabolic pathway levels was calculated using Spearman's correlation analysis.

### Construction of Gene–Metabolite Interaction Network and Identification of the Most Discriminatory Metabolite for Metastatic Niche

Marker genes of Malig‐5 and Neutro‐0/Neutro‐3 with fold change > 1 and *p* < 0.05 were selected and then separately intersected to the abovementioned EMT and NET gene sets. Marker metabolites of enriched pathways within metastatic niches were selected with a VIP value of >0.5, *p* < 0.05, and a fold change of >0. These genes, metabolites, and identified ligand–receptor pairs were uploaded to MetaboAnalyst 6.0 to build a gene–metabolite interaction network that played a role in driving metastasis. Subsequently, the discriminating abilities of marker metabolites between metastatic niches and other regions were evaluated and ranked through three machine learning algorithms, including Boruta, Random Forest, and Xgboost based on the R package of “Boruta” (v 8.0.0), “randomForest” (v 4.7.1.1), and “xgboost” (v 1.7.7.1). The most discriminatory metabolites were then identified.

### Investigation of the Pro‐Metastasis Role for Palmitic Acid in NSCLC and Pan‐Cancer

Palmitic acid exhibited the strongest ability to distinguish metastatic niches from those in other regions. To investigate the role of palmitic acid in metastasis, the levels of palmitic acid in the blood of patients were first compared with NSCLCs with and without BMs using metabolic mass spectrometry data from the QMH‐NSCLC cohort. Next, the spatial metabolomics and transcriptomics slides were divided into high and low palmitic acid regions according to the 50% median palmitic acid expression. DEGs between these two regions, with the low palmitic acid region serving as a control group, were identified using the “FindAllMarkers” function under the criteria of min.pct > 0.25, logFC > 0.25, and adjusted *P* < 0.05. The top 200 upregulated DEGs were imported into Metascape (https://metascape.org/gp/index.html#/main/step1) for GO enrichment analysis. Subsequently, palmitic acid and these DEGs were uploaded to MetaboAnalyst 6.0 to construct a palmitic acid–gene regulatory network. The palmitic acid regulatory scores were then calculated in the pan‐cancer cohort using the GSVA method of the R package “GSVA” (v 1.50.5), based on the palmitic acid regulatory genes identified above. Regulatory scores for palmitic acid were compared between patients with and without metastases (including lymph node and distant metastases).

### Virtual Drug Screening, Molecular Docking Targeting, and Therapeutic Response Analysis Targeting Palmitic Acid

The direct targeting of palmitic acid with drugs was challenging; however, some medications could inhibit fatty acid biosynthesis. Accordingly, 13 FASN inhibitors were selected from previous studies^[^ ^28^, ^29^, ^69^ ^]^ and downloaded the structural data of these small‐molecule drugs from the ZINC database (https://zinc20.docking.org/) and DrugBank database (https://go.drugbank.com/), separately. FASN‐ERD was a crucial protein domain responsible for palmitic acid synthesis,^[^ ^70^ ^]^ and its structural data were retrieved from the Protein Data Bank (https://www1.rcsb.org/). The interactions between small‐molecule drugs and FASN‐ERD were analyzed using the PyRx software (v 0.8). Specifically, a receptor grid file was prepared using the AutoDock Wizard module, ligand molecules were processed using the open Babel module, and virtual screening was conducted using the Vina and AutoDock Wizard modules to determine the lowest binding energy conformation for each drug. The binding pocket was visualized in 3D using the PyMol software (v 1.8), and intermolecular interactions were shown in 2D using the MOE software (v 2015). Moreover, experimental and clinical evidence for the anticancer effects of these drugs was comprehensively obtained from published studies and the ClinicalTrials.gov platform (https://clinicaltrials.gov/). The drug sensitivities of the high and low palmitic acid regions were calculated and compared using the R package “Beyondcell” (v 2.2.0), an algorithm specifically designed to assess the heterogeneity of cancer therapeutic responses.

### Statistical Analysis

Statistical analyses were performed using R (v. 4.3.1), Python (v. 3.11), GraphPad Prism (v. 8.0), and SPSS (v. 26.0). Unless specified otherwise, groups were compared using either the Wilcoxon rank‐sum test (for two groups) or the Kruskal–Wallis test (for three or more groups). Spearman's rank correlation was used to assess variable relationships. All tests were two‐sided, and statistical significance was set at *p* < 0.05.

## Conflict of Interest

The authors declare no conflict of interest.

## Author Contributions

B.C., K.M.K., and F.L. are contributed equally to this work. L.Z., G.K.‐K.L., L.Z., and T.S. conceived of designed, and supervised the study and surgery. B.C., K.M.K., F.L., J.S., E.D., H.Z., X.F., J.L., and L.Z. developed and performed the experiments or collected data. B.C., L.Z., C.W., E.D., and J.L. designed and performed the computation and statistical analyses. C.L., X.L., G.X., T.S., and L.Z. performed surgical operations. All authors have written, reviewed, and edited the manuscript.

## Supporting information



Supporting Information

## Data Availability

The data that support the findings of this study are available on request from the corresponding author. Codes used in this study are available in the Google Drive (https://drive.google.com/drive/folders/1yIeZ4a1BsBHl2CPrswVpKOhwuhJaTkFX?usp=drive_link).
