# Supplementary material for: Neutrophil Extracellular Trap Reprograms Cancer Metabolism to Form a Metastatic Niche Promoting Non‐Small Cell Lung Cancer Brain Metastasis
Source: Adv Sci (Weinh). 2025 Nov 18;13(5):e08478. doi: 10.1002/advs.202508478 (PMC12850224; doi:10.1002/advs.202508478)
Supplement: Supplementary file 1 — Supporting Information [file ADVS-13-e08478-s001.docx]

**Supporting Information for**

**“Neutrophil extracellular trap reprograms cancer metabolism to form metastatic niche promoting non-small cell lung cancer brain metastasis”**

By Chen et al.

**1. Experimental Section**

*1.1. Copy number variation (CNV) estimation and identification of malignant cells and malignant spatial regions*

To distinguish malignant cells from epithelial cells, we employed the InferCNV algorithm^1^ to estimate CNVs. At the single-cell level, the R package ‘inferCNV’ (v 1.16.0) was utilized, with endothelial cells as the normal reference^2^ and the cut-off value of 0.1. We scored each cell using two CNV parameters^3^: (1) CNV scores deflecting the overall CNV signal were calculated by taking the mean of the squares of the CNV values across the genome. (2) After ranking epithelial cells by CNV values, the CNV correlation was defined as the Spearman correlation coefficient between each cell's CNV and the average CNV of the top 5% of ranking epithelial cells. Cells with a CNV score > 0.6 and a CNV correlation exceeding 0.2 were classified as putative malignant cells. At the spatial level, the R package ‘SPATA2’ (v 2.0.4) was used to compute the CNA scores for each spot. The fraction of malignant cells in each spot was estimated using the python package ‘cell2location’ (v 0.1.3) with cell annotations from the paired single-cell RNA sequencing (scRNA-seq) samples. Next, the spatial distribution consistency between the CNA scores and malignant cell fractions was examined to assess the accuracy of identifying malignant cells and regions.

*1.2. Identification of DEGs and gene regulatory networks across cell subpopulations*

Malignant cells and immune cells (neutrophils, macrophages, and T cells) were selected for reclustering to identify cell subpopulations, with optimal cluster resolutions of 0.3 for malignant cells and 0.5 for immune cells, determined using the R package ‘clustree’ (v0.5.1). Next, we used the ‘FindAllMarkers’ function to identify DEGs for each subpopulation, under the criteria: min.pct > 0.25，logFC > 0.25, and adjusted *p* < 0.05. We also employed the R package ‘SCENIC’ (v 1.2.4) to infer gene regulatory networks (transcription factor regulon) based on the motif database for RcisTarget. Specifically, the regulon activity for each cell was scored using the R package ‘AUCell’ (v 1.24.0), and the regulon specificity score for each subpopulation was calculated based on Jensen–Shannon divergence (JSD) to determine the subpopulation-specific regulon, following a previously established method^4^.

*1.3. Cellular gene set enrichment analysis and metabolic activity evaluation*

To understand the cellular functions in each subpopulation of malignant cells and neutrophils, we conducted the enrichment analyses using the AUCell, singscore, and ssGSEA rank-based scoring methods of the R package ‘irGSEA’ (v 3.3.2), based on scRNA-seq data matrix. Hallmark gene sets were collected from the Molecular Signatures Database (MSigDB)^5^. Three individual gene sets were retrieved: (1) Epithelial-mesenchymal transition (EMT) genes from MSigDB: M5930 and Tagliazucchi et al^6^; (2) Neutrophil extracellular trap (NET) genes from Xu et al^7^; and (3) long-chain fatty acid biosynthesis genes annotated in GO:0042759. Next, the metabolic pathway activity in each subpopulation of malignant cells and neutrophils was quantified following the computational pipeline established by Xiao et al.^8^. Briefly, metabolism gene lists and pathways were curated from the Kyoto Encyclopedia of Genes and Genomes (KEGG) database. The pathway activity score was determined by calculating the weighted average of the relative gene expression across different cell subpopulations, and statistical significance was assessed using permutation tests. A score > 1 or < 1 indicates that the activity level is significantly higher or lower than the average, respectively, in the specific cell subpopulation.

*1.4. Metastasis-initiating cell (MIC) validation*

MIC was validated in the single-cell RNA sequencing (scRNA-seq) dataset GSE131907. The alignment of malignant cell subclusters between GSE131907 and HKU-NSCLC was performed with the 'cellhint' Python package (v1.0.0). Next, pseudotime analysis was conducted using CytoTrace, Monocle2, and VIA tools. EMT levels were assessed through the AUCell, singscore, and ssGSEA rank-based scoring methods of the R package ‘irGSEA’ (v 3.3.2).

*1.5. Cancer immunity cycle, immune immunomodulator, and immunocyte proportion analyses*

Patients with TCGA-NSCLC were divided into two groups (Malig-5^high^ and Malig-5^low^) using the median Malig-5 proportion as a cutoff. The cancer immune cycle comprises seven major steps that reflect the infiltration of immune cells^9^. The activities of these steps were quantified in TCGA datasets by Xu et al. using the ssGSEA algorithm^10^. Next, we compared the differences in the activity of these steps between Malig-5^high^ and Malig-5^low^ NSCLC tumors. Immunomodulators are essential for regulating the immune response. Seventy-eight immunomodulators were gathered from a previous study^11^, including co-stimulators, co-inhibitors, ligands, receptors, cell adhesion molecules, antigen-presenting molecules, and other categories. Gene expression levels of these immunomodulators were compared between Malig-5^high^ and Malig-5^low^ NSCLC tumors. The correlation between the fraction of Malig-5 cells and the proportions of other cell types was calculated using Spearman’s correlation analysis.

*1.6. Spatial enrichment analysis*

To estimate the spatial distribution of EMT and NET in spatial transcriptomics slides, we computed the enrichment score for each spot using the GSVA and ssGSEA methods of the R package ‘GSVA’ (v 1.50.5), with the EMT and NET gene sets mentioned above. Specifically, the EMT gene set was obtained from the ‘HALLMARK_EPITHELIAL_MESENCHYMAL_TRANSITION’ (MSigDB: M5930), whereas the NET gene set was sourced from another earlier study^7^. The distribution of EMT and NET in each slide was visualized using the ‘SpatialFeaturePlot’ function.

*1.7. Within-class neighborhood analysis*

Malig5 and Neutro-0/Neutro-3 exhibited high spatial colocalization. To further assess the extent of spatial clustering between Malig-5 and Neutro-0/Neutro-3, we performed within-class neighborhood analysis (homotypic score calculation) according to the method proposed by Bäckdahl et al^12^. The Cell2location deconvolution matrix and spatial coordinate information were used as input files. The neighboring relationships of each spot were identified using the ‘kNN’ function of the R package ‘dbscan’ (v 1.1.12), and then spatial networks were generated. Spots with a proportion of Malig-5 or Neutro0/Neutro-3 > 0.5 were separately screened as Malig-5^high^ spots or Neutro0/Neutro-3^high^ spots, respectively. Based on the spatial networks, the cell degree (the number of directly adjacent spots) was calculated for the adjacent Neutro0/Neutro-3^high^ spots of Malig-5^high^ spots, and was visualized in each slide using the R package of ‘ggplot2’ (v 3.4.4). The degree ranges from a minimum of 0 to a maximum of 6.

*1.8. Cell transfection*

Both KRT10-targeting small interferinig RNA (siRNA) and negative control siRNA were supplied by RIBOBIO (siBDM1999A, RIBOBIO, China,), and were then transfected into the A549, BM101 (details regarding BM101 refer to previous publication^13^), and PC9 cell lines via Lipofectamine 3000 (Thermo Fisher Scientific, USA). Twenty-four hours after transfection, cells were collected for other assays.

*1.9. In vitro drug treatment and Transwell assay*

Palmitic acid (S3794, Selleck, USA) and TVB-2640 (HY-112829, MCE, USA) stock solutions were dissolved in DMSO, then reconstituted in base MEM medium to final concentrations of 50 μM and 0.1 μM respectively. Cells were treated with palmitic acid and/or TVB-2640 for 24 hours.

Cell migration and invasion were evaluated with uncoated and Matrigel-coated Transwell inserts (8-μm pores; BD Biosciences, USA) respectively. For migration assays, 2×10⁴ cells in serum-free medium were seeded in the upper chamber, with 20% FBS-DMEM as chemoattractant below. After 36-hour incubation, migrated cells on the membrane underside were fixed with 100% methanol, stained with 0.5% crystal violet, and quantified. Invasion assays followed identical procedures but used Matrigel-coated inserts and 5×10⁴ cells, with traversed cells representing invasive potential.

*1.10. Immunofluorescence analysis*

The cultured A549, BM101, and PC9 cells were fixed with 4% paraformaldehyde and blocked with 5% BSA. Next, the cells were treated with specific primary antibodies (LOX, 1:300, F2157, Selleck, China; Phallodin, 1:200, RM02835, ABclonal, China) at 4°C overnight and corresponding secondary antibodies at 37°C for 1 hour. The cells were then counterstained with DAPI for 10 minutes and imaged under a confocal microscopy (LSM 880, ZEISS, Germany).

*1.11. Protein extraction and western blotting*

Cells were lysed using RIPA lysis buffer (9806, CST, USA) with protease and phosphatase inhibtor (A32961, Thermo Scientific, USA). Protein lysates were loaded onto SDS-PAGE gels and subjected to electrophoresis. The separated proteins were transferred to PVDF membranes and blocked with a 5% non-fat milk solution in 5% TBST for 1 hour. For immunodetection, primary antibodies of Fibronectin (1:1000, 26836, CST, USA), E-Cadherin (1:1000, 9782, CST, USA), and GAPDH (1:2000, 2118, CST, USA) were used. Antibody binding was detected by enhanced chemiluminescent HRP substrate detection kit (WBKLS0500, Millipore, USA). Bands were exposed by Amersham Imager 680 blot and gel imager (GE Healthcare, USA).

*1.12. Human Cerebral organoids culture*

HiPSCs were seeded and cultured in mTeSR™1 medium (STEMCELL Technologies, Canada) on Matrigel, according to the protocol reported elsewhere. Human iPSCs were trypsinized and 9×10^3^ cells were plated in each well of an ultra-low 96-well U-bottom plate (Corning Costar, USA) in hESC medium, with every 10 ml containing 8 ml of DMEM-F12 (11320082, Invitrogen, USA), 2 ml of KOSR (A3181502, Invitrogen, USA), 0.3 ml of ESC-quality FBS (10439016, Gibco, USA), 0.1 ml of GlutaMAX (35050061, Invitrogen, USA), 0.1 ml of MEM-NEAA (M7145, Sigma, USA), 0.07 ml of 2-mercaptoethanol (805740, Merck, Germany), 1🞨 penicillin/streptomycin, and 4 ng/ml bFGF (233-FB, R&D Systems, USA). On days 5 to 6, half of the medium was gently replaced with induction medium, every 10 ml of which consisted of 8 ml of DMEM-F12 (11320082, Invitrogen, USA), 1🞨 N2 supplement (17502001, Invitrogen, USA), 2 ml of KOSR (A3181502, Invitrogen, USA), 0.3 ml of ESC-quality FBS (10439016, Gibco, USA), 1🞨GlutaMAX (35050061, Invitrogen, USA), 1🞨 MEM-NEAA (M7145, Sigma, USA), 0.07 ml of 2-mercaptoethanol (805740, Merck, Germany), 1🞨 Penicillin/Streptomycin, 4 ng/ml WNT-3A (5036-WN, R&D Systems, USA), 1 µM CHIR99021 (C2447-2s, Cellagentech, USA), and 1 µM SB-431542 (C7243-5, Cellagentech, USA). On day 8, organoids were embedded in Matrigel (BD Biosciences, USA) and continued to grow in induction medium for three more days. On day 11, embedded organoids were plated on an Orbital shaker at a speed of 80 rpm/min and grown in cerebral organoid differentiation medium. Every 10 ml of this medium consisted of 5 ml of DMEM-F12 (11320082, Invitrogen, USA), 5 ml of Neurobasal medium (21103049, Invitrogen, USA), 0.05 ml of N2 supplement (17502001, Invitrogen, USA), 0.5 ml of insulin (1544-IR-050, R&D Systems, USA), 0.1 ml of GlutaMAX supplement (35050061, Sigma, USA), 0.05 ml of MEM-NEAA (M7145, Invitrogen, USA), 0.1 ml of B27 supplement (17504044, Invitrogen, USA), and 0.1 ml of penicillin–streptomycin (10378016, Invitrogen, USA).

*1.13. Neutrophil NETs and NSCLC cell co-culture in cerebral organoids*

Cerebral organoids were microinjected on day 30-40 after seeding into 60 μl Matrigel in a 24-well culture plate. Microinjection of tumor cells into human cerebral organoids was completed within 60 min of tumor resection or biopsy. Tumors cells were trypsinized into single cells and suspended in Advanced DMEM-F12 at a density of 1×10^5^/μl. Neutrophil were isolated as described in the main text, and cultured in RPMI 1640 medium supplemented with 10% fetal bovine serum (FBS, Gibco, USA) and 1% penicillin-streptomycin antibiotics at 37°C in a 5% CO2 incubator. After stimulating neutrophils with Phorbol-12-myristate-13-acetate (PMA, p8129-5MG, Sigma-Aldrich, USA), NETs were underwent QD labelling using Qtracker 625 Cell Labelling Kit (Invitrogen, USA) according to the manufacturer's protocol. BM101 siNC and siKRT10 were undervent QD labelling using Qtracker 525 Cell labelling Kit (Invitrogen, USA) as the protocol. Tumor cells and neutrophil NETs were microinjected (10-μl) into organoids using a 26-gauge Hamilton gastight 1701 syringe under stereomicroscope (MZ75, Leica, Germany). The organoids were monitored daily under bright-field or fluorescence microscopy post-injection before being sent for immunofluorescence analysis and single-cell sequencing.

*1.14. Animal experiments*

Six weeks-old BALB/cAnN-nu mice were purchased from Hunan SJA Laboratory Animal Co., Ltd (Changsha, China), and were housed in a mouse-specific pathogen-free facility for at least one week before starting the downstream processes. A549 cells (1🞨10^6^) were suspended in 100 μL of serum-free DMEM and then injected into the tail vein. Tumor growth and metastasis was assessed by bioluminescence measurements using the PE-IVIS spectrum in vivo imaging system (PerkinElmer, USA) following intraperitoneal injection of the reporter substrate D-luciferin (15 mg/ml, LUCK-100, GoldBio, USA). On day 21 post-injection, tumor-bearing mice were randomized based on tumor size to ensure comparable groups, and then treated with vehicle (10% DMSO, 40% PEG300, 5% Tween-80, and 45% saline), palmitic acid (5 mg/kg, S3794, Selleck, USA), or TVB-2640 (10 mg/kg, HY-112829, MCE, USA). All treatments were administered daily via intraperitoneal injection. The animal experiments were designed using two independent cohorts: one for in vivo imaging and sample collection (*n* = 4 per group), and the other for evaluating overall survival (*n* = 5 per group).

**2. Figures**


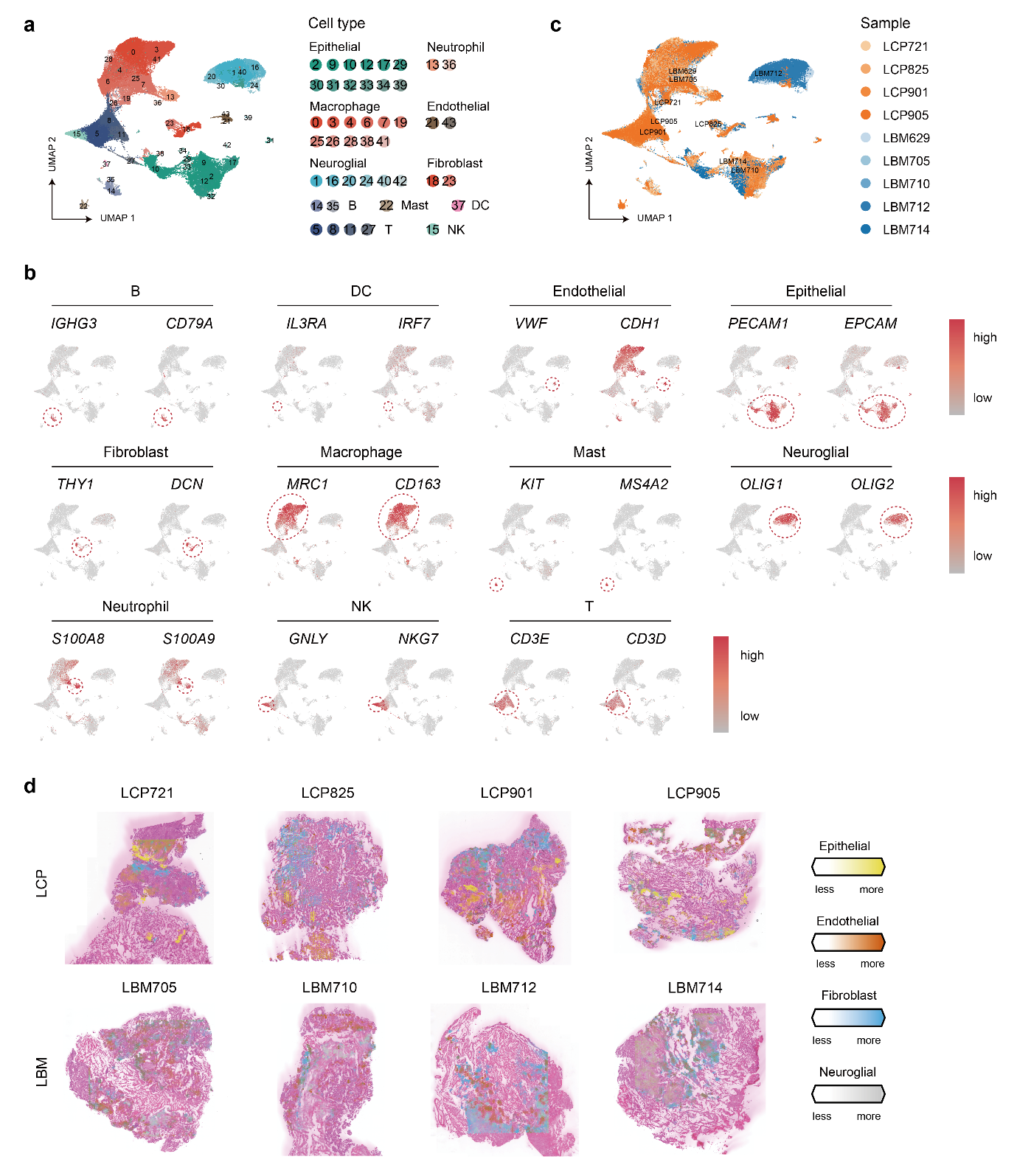


**Figure S1.** Cellular and molecular characterization of primary non-small cell lung cancer (NSCLC) and brain metastasis (BM) tissues using single-cell RNA sequencing (scRNA-seq) and spatial transcriptomics analyses. Related to Figure 1. a) Uniform manifold approximation and projection (UMAP) plot of 94,383 cells colored by the cell clusters in the XY-NSCLC scRNA-seq data. b) UMAP plot showing the expression of canonical marker genes presented in Figure 1c for each cell type. c) UMAP plot of 94,383 cells colored by the tissue samples. d) Visualization of the spatial distribution of stromal cell types on LCP and LBM spatial transcriptomics slides, deconvoluted using Cell2location.


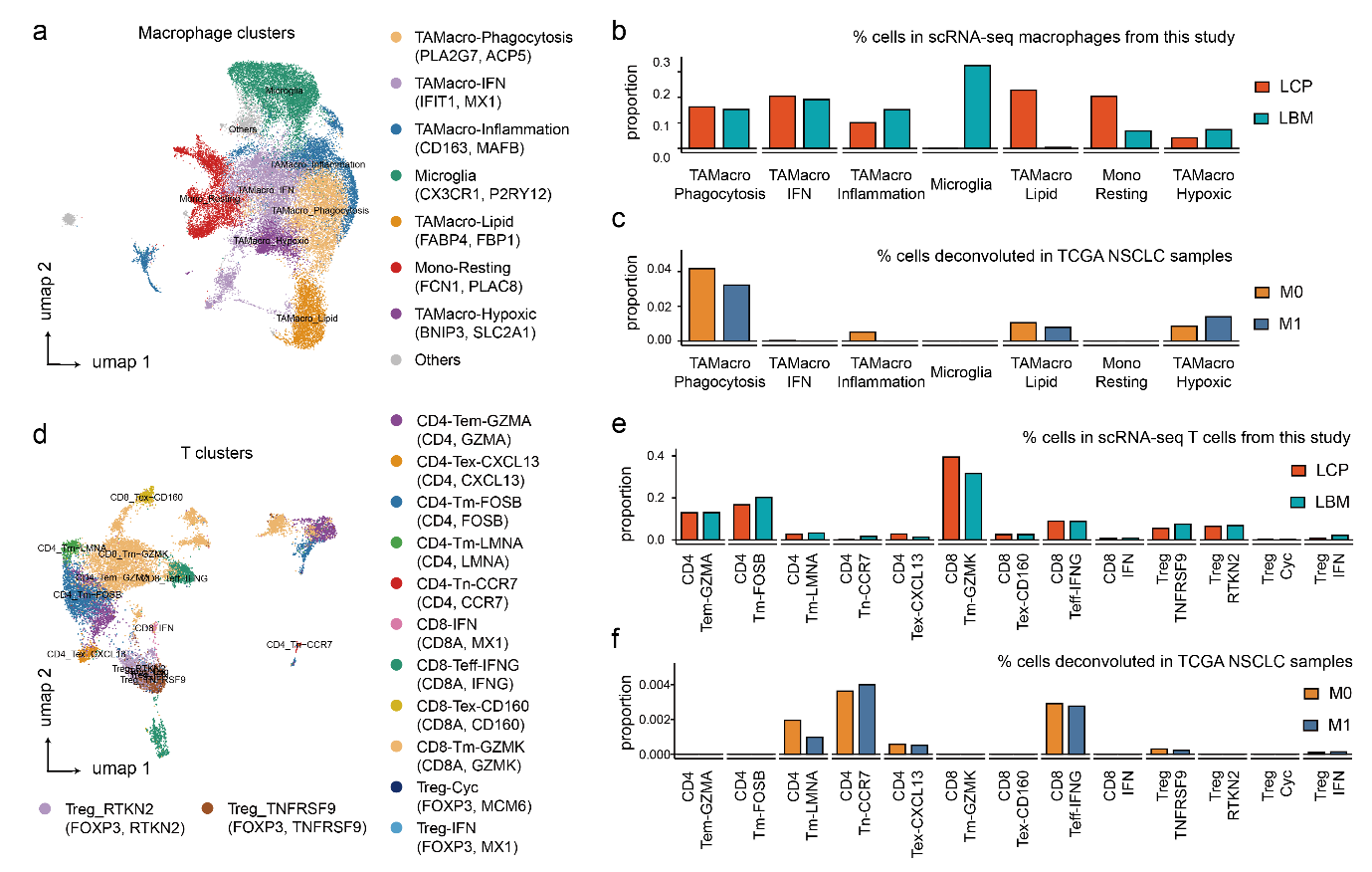


**Figure S2.** Subcluster characterization of macrophage and T cells in primary NSCLC and BM tissues using scRNA-seq analysis. a) UMAP plot showing 8 macrophage subclusters in lung cancer primary (LCP) and lung cancer BM (LBM) tissues from XY-NSCLC cohort. Cells were colored by subclusters. b) Bar plots comparing macrophage subcluster abundance between LCPs and LBMs based on the XY-NSCLC scRNA-seq data. c) Bar plots comparing macrophage subcluster abundance between LCPs with metastasis (M1) and without metastasis (M0) using deconvoluted bulk RNA-seq data from the TCGA NSCLC cohort. d) UMAP plot showing 13 T subclusters in LCP and LBM tissues from XY-NSCLC cohort. Cells were colored by subclusters. e) Bar plots comparing T subcluster abundance between LCPs and LBMs based on the XY-NSCLC scRNA-seq data. f) Bar plots comparing T subcluster abundance between LCPs with metastasis (M1) and without metastasis (M0) using deconvoluted bulk RNA-seq data from the TCGA NSCLC cohort.

**
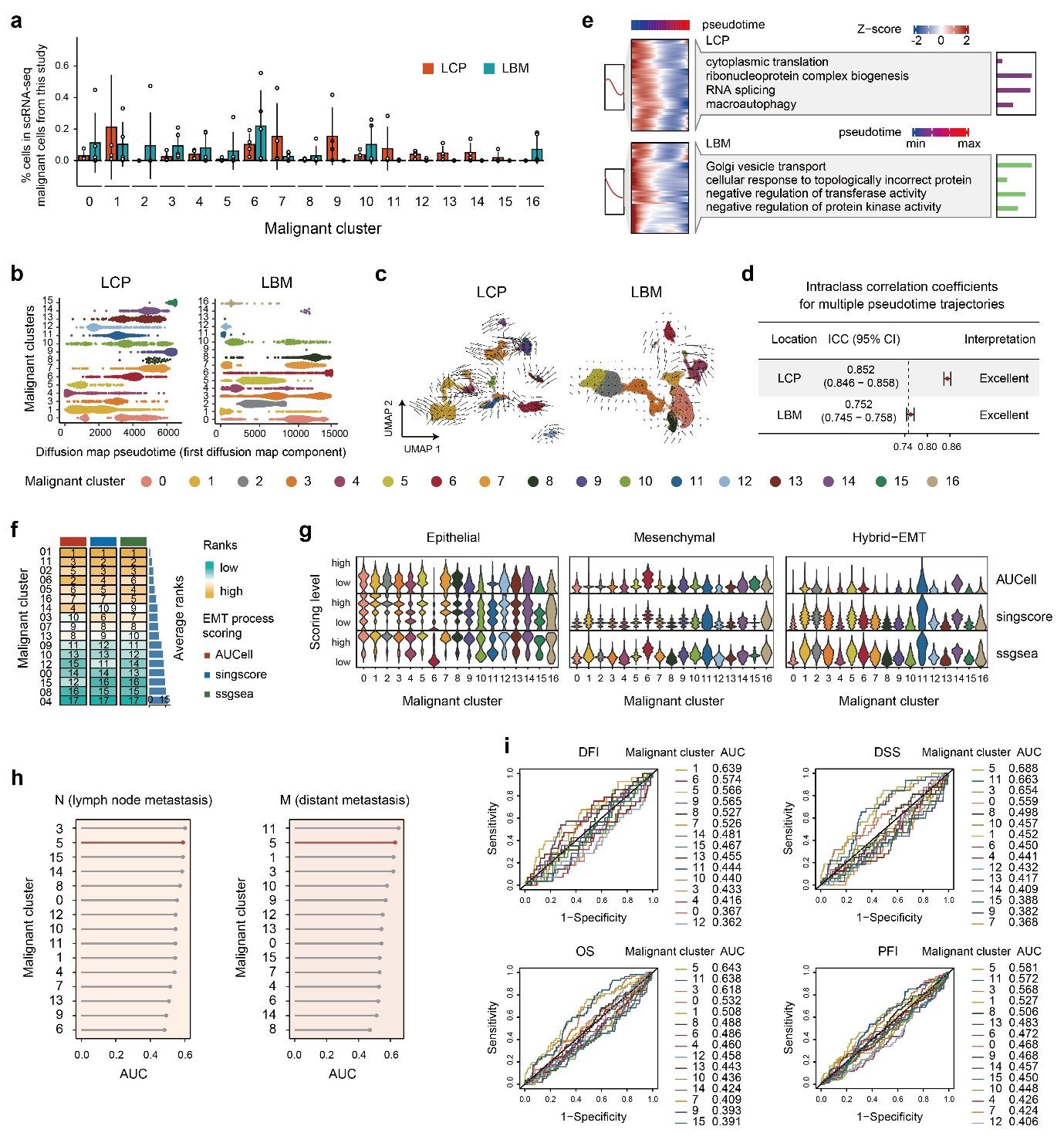
**

**Figure S3.** Differentiation trajectories of malignant cells define the metastasis-initiating cell (MIC) *LOX*^+^ Malig-5. Related to Figure 2. a) Bar plots comparing the abundance of malignant cell subclusters between LCPs and LBMs based on the XY-NSCLC scRNA-seq data. b) Pseudotime trajectory plots of malignant cell subclusters in LCPs (left) and LBMs (right), inferred by diffusion map. c) RNA velocity plot showing cell differentiation directions among malignant cell subclusters in LCPs (left) and LBMs (right). d) Intraclass correlation coefficient (ICC) analysis evaluating the reliability of four pseudotime trajectories: CytoTRACE, Monocle2, diffusion map, and PyVIA. e) Heatmap showing declined gene expression patterns in malignant cell subclusters with corresponding GO functional enrichment along pseudotime (top: LCPs; bottom: LBMs). f) Ranks of ‌epithelial-mesenchymal transition‌ (EMT) process (HALLMARK: M5930) scores of malignant cell subclusters in each quantification algorithm of AUCell, singscore, and ssGSEA. g) Violin plot showing EMT status (epithelial, hybrid EMT, and mesenchymal) scores, as proposed by Tagliazucchi et al.^6^, quantified using ssGSEA, AUCell, and singscore for each malignant cell subcluster. h) Ranking of malignant cell subclusters based on AUC values for predicting distant or lymphatic metastasis in the TCGA NSCLC cohort. The red line highlights *LOX*^+^ Malig-5, which exhibits a strong ability to predict metastasis. i) Receiver operating characteristic (ROC) curves for survival prediction (including DFI, DSS, OS, and PFI) using the proportion of malignant cell subclusters in the TCGA NSCLC cohort. ‌DFI‌, ‌Disease free interval; DSS, ‌Disease specific survival; ‌OS‌, Overall survival; PFI‌, Progression free interval.‌


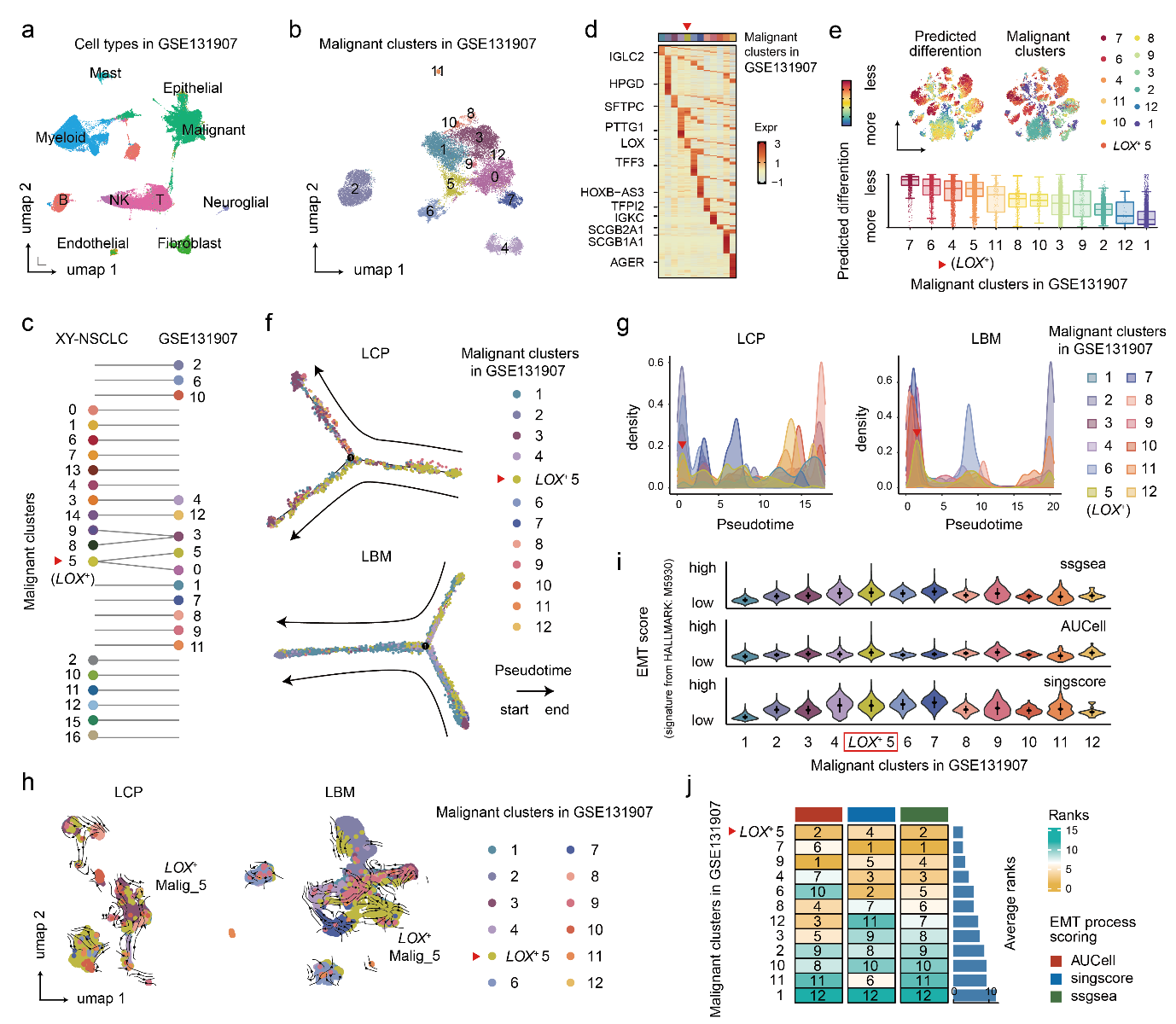


**Figure S4.** Validation of *LOX*^+^ Malig-5 as MICs. a) UMAP plot showing the integrated single-cell map of 11 LCP and 10 LBM samples, originating from GSE131907. Cells were colored by cell types. b) UMAP plot showing 13 subclusters of malignant cells from LCPs and LBMs. Cells were colored by subclusters. c) Cellhint harmonization graph illustrating cell-cluster relationships between the XY-NSCLC training dataset and GSE131907 validation dataset. In GSE131907, Malig-0 and Malig-5 directly correspond to *LOX*^+^ Malig-5 in XY-NSCLC. Thus, Malig-0 and Malig-5 in GSE131907 were renamed as *LOX*^+^ Malig-5. Note that the lines in the graph reflect the connections among transcriptionally similar cell types regardless of their names. d) Heatmap showing the expression of marker genes across malignant cell subclusters. e) T-distributed stochastic neighbor embedding (TSNE) plots (top) and bar plots (bottom) displaying differentiation states for each malignant cell subcluster using CytoTRACE. f) Monocle pseudotime trajectory of malignant cell subclusters in LCPs (top) and LBMs (bottom), inferred by Monocle2. g) Cell density distribution of malignant cell subsubtypes along with the pseudotime in LCPs (top) and LBMs (bottom). Arrows refer to MIC *LOX*^+^ Malig-5. h) VIA pseudotime trajectory model of malignant cell subclusters in LCPs (left) and LBMs (right), inferred by PyVIA. i) Violin plot showing EMT process (HALLMARK: M5930) scores, quantified by ssGSEA (top), AUCell (middle), and singscore (bottom) for each malignant cell subcluster. j) Ranks of EMT process (HALLMARK: M5930) scores of malignant cell subclusters in each quantification algorithm of AUCell, singscore, and ssGSEA. Except where noted, MIC *LOX*^+^ Malig-5 was highlighted by red boxes or triangles.


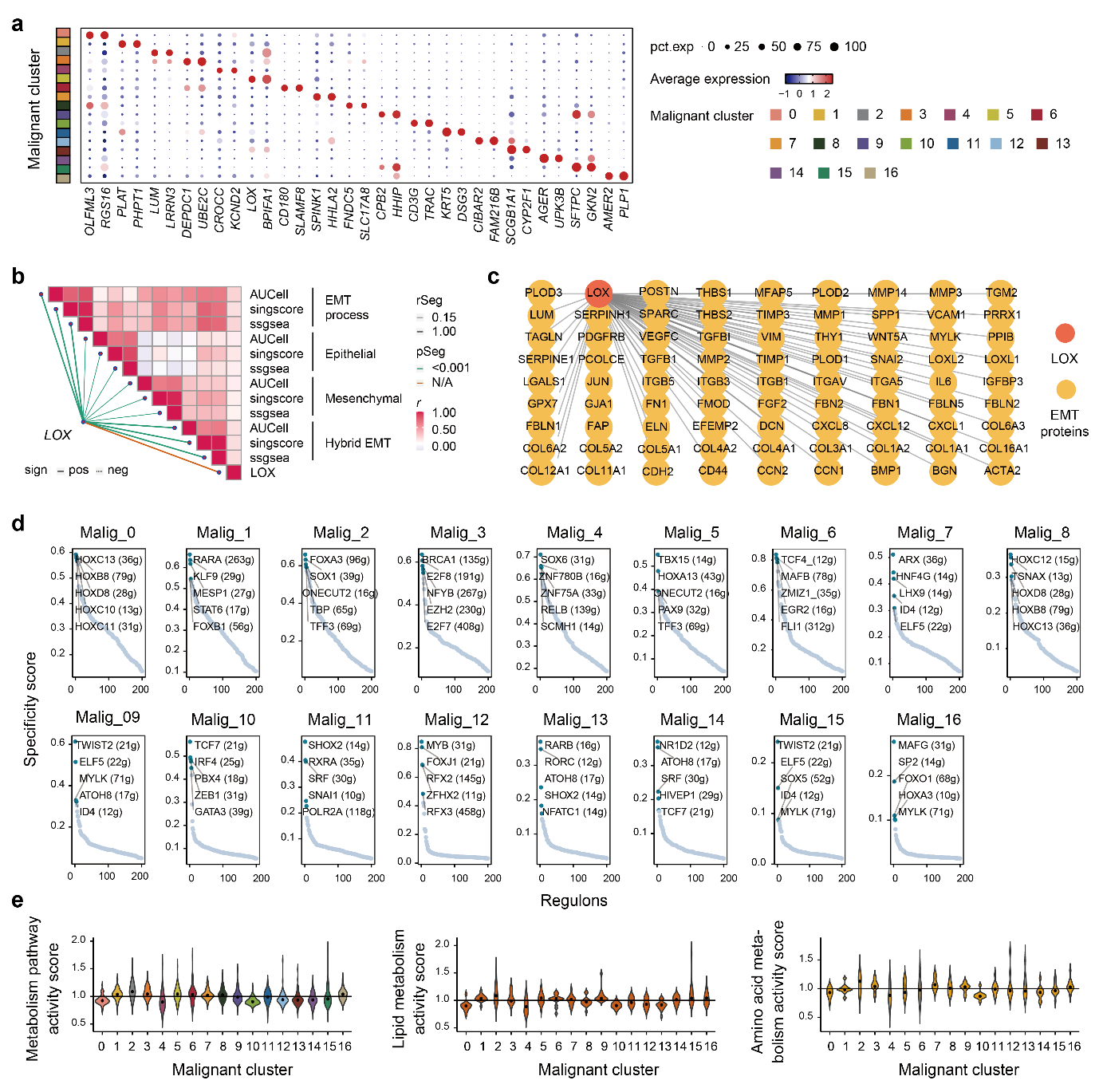


**Figure S5.** Molecular and metabolic pathway characteristics of malignant subclusters. Related to Figure 2. a) Dot plot displaying marker genes across malignant cell subclusters in the XY-NSCLC scRNA-seq data. Dot size indicates the proportion of cells expressing the specific genes. Color intensity represents the average scaled expression level of specific genes. b) Correlations between *LOX* expression and EMT process and status (epithelial, mesenchymal, hybrid EMT) scores in malignant cells. The scores were quantified by AUCell, singscore, and ssGSEA, and the correlations were analyzed using Spearman correlation. c) Protein-protein interaction (PPI) network showing the interaction between LOX and EMT proteins. d) Top 5 differential activated transcription factors in each malignant cell subcluster, identified by SCENIC analysis. e) Violon showing Kyoto Encyclopedia of Genes and Genomes (KEGG) metabolic pathway (including overall metabolism, lipid metabolism, amino acid metabolism) activity scores in each malignant cell subcluster, inferred from scRNA-seq data.


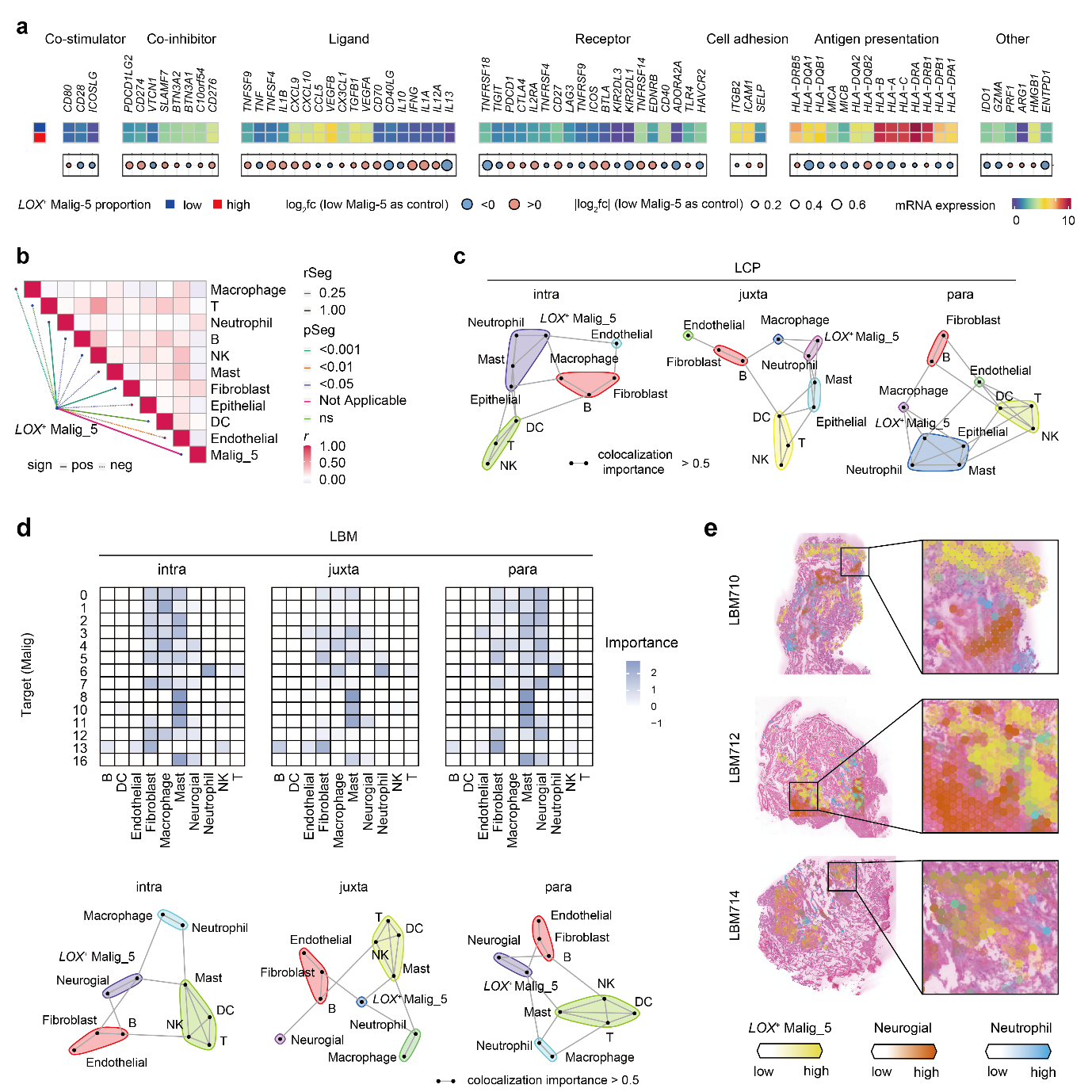


**Figure S6.** *LOX*^+^ Malig-5 colocalizes with the neutrophils. Related to Figure 3. a) Heatmap showing the expression disparities of immune modulator molecules between NSCLC samples with high and low *LOX*^+^ Malig-5 proportions in the TCGA NSCLC cohort. b) Correlation between the cellular proportion between *LOX*^+^ Malig-5 and other cell types. The correlation was analyzed using Spearman correlation. c) Network community plots showing the colocalization relationship between *LOX*^+^ Malig-5 and other cell types in the multiple neighborhood views (including intrinsic, juxta, and para) of LCP spatial transcriptomics slides. d) Heatmap (top) and network community (bottom) plots exhibiting the colocalization relationship between *LOX*^+^ Malig-5 and other cell types in the multiple neighborhood views (including intrinsic, juxta, and para) of LBM spatial transcriptomics slides. The lines connecting cells represent strong colocalizations, with an importance > 0.5. e) Visualization of the spatial distribution of *LOX*^+^ Malig-5 and neutrophils on LBM spatial transcriptomic slides by Cell2location.


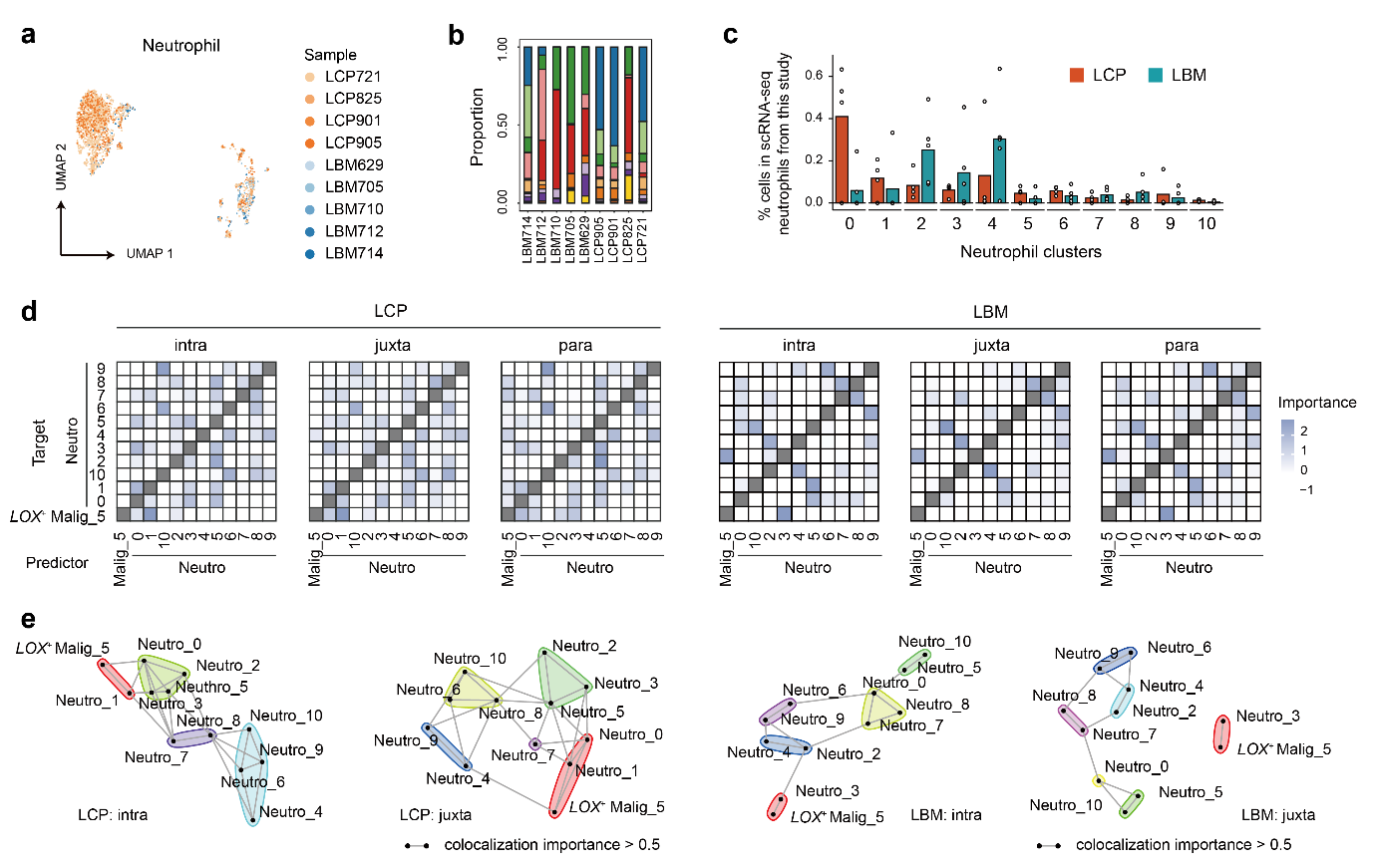


**Figure S7.** *LOX*^+^ Malig-5 colocalizes with the neutrophil subtypes Neutro-0 and Neutro-3. Related to Figure 3. a) UMAP plot of neutrophils colored by the tissue samples in the XY-NSCLC scRNA-seq data. b) Stacked bar plot showing the proportions of neutrophil subclusters across LCP and LBM samples. c) Bar plots comparing the abundance of neutrophil subclusters between LCPs and LBMs. d) Heatmap showing the colocalization relationship between *LOX*^+^ Malig-5 and neutrophil subclusters in the multiple neighborhood views (including intrinsic, juxta, and para) of LCP (left) and LBM (right) spatial transcriptomics slides. e) Network community showing the colocalization relationship between *LOX*^+^ Malig-5 and neutrophil subclusters in the multiple neighborhood views (including intra and juxta) of LCP (left) and LBM (right) spatial transcriptomics slides. The lines connecting cells represent strong colocalizations, with an importance greater than 0.5.


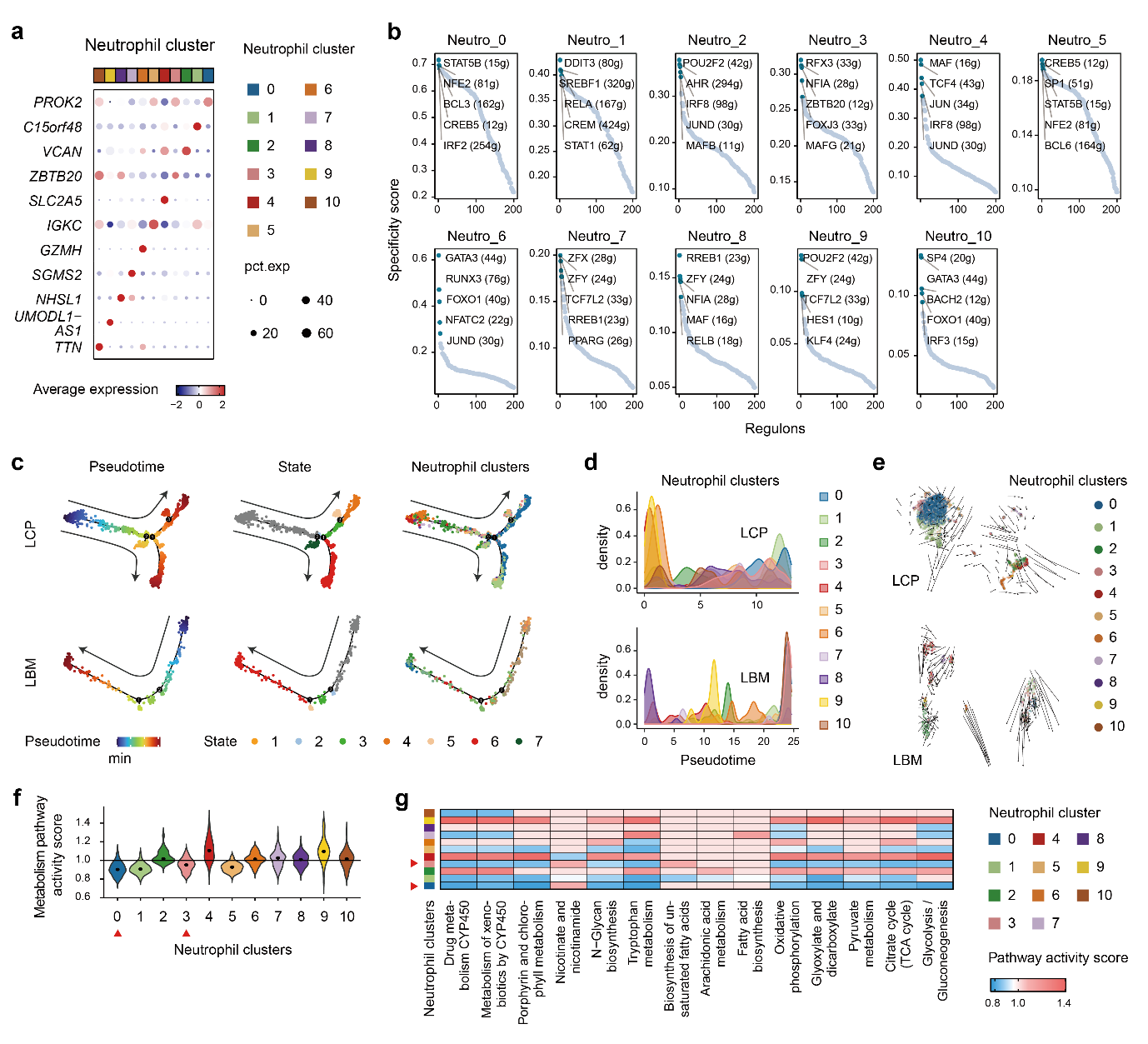


**Figure S8.** Molecular, differentiation, and metabolic characteristics of neutrophil subtypes Neutro-0 and Neutro-3. Related to Figure 3. a) Dot plot displaying marker genes across neutrophil subclusters in the XY-NSCLC scRNA-seq data. Dot size indicates the proportion of cells expressing the specific genes. Color intensity represents the average scaled expression level of specific genes. b) Top 5 differential activated transcription factors in each neutrophil cluster, identified by SCENIC analysis. c) Monocle pseudotime trajectory of neutrophil subclusters in LCPs (top) and LBMs (bottom), inferred by Monocle2. Trajectory is colored by pseudotime (left), cell states (middle), and neutrophil subclusters (right). d) Cell density distribution of neutrophil subclusters along with the pseudotime in LCPs (top) and LBMs (bottom). e) RNA velocity plot showing cell differentiation directions among neutrophil subclusters in LCPs (top) and LBMs (bottom). f) Violon showing overall KEGG metabolic pathway activity scores in each neutrophil subcluster, inferred from scRNA-seq data. g) Heatmap visualizing specific KEGG metabolic pathway activities across neutrophil subclusters, inferred from scRNA-seq data.


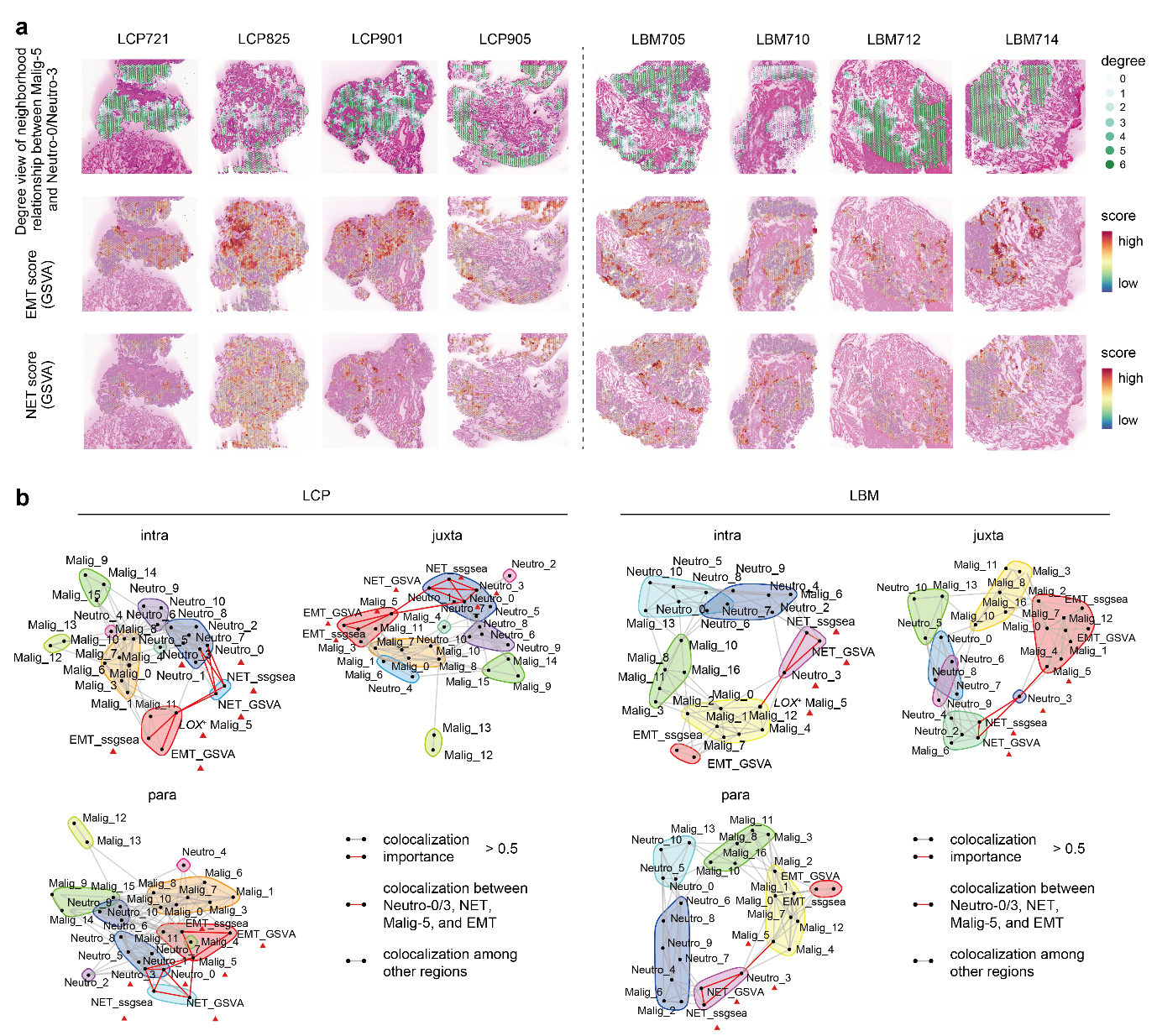


**Figure S9.** Neutro-0 and Neutro-3 are co-localized with *LOX*^+^ Malig-5. Related to Figure 4. a) Top, visualization of the cell degree (the number of directly adjacent spots) between Neutro-0/Neutro-3^high^ and Malig-5^high^ spots on LCP and LBM spatial transcriptomics slides. Bottom, visualization of EMT and neutrophil extracellular trap (NET) scores, quantified using the GSVA algorithm. b) Network community showing the colocalization relationship between malignant cell subclusters, neutrophil subclusters in the multiple neighborhood views (including intra, juxta, and para). The lines connecting cells represent strong colocalizations with an importance larger than 0.5, and *LOX*^+^ Malig-5, Neutro-3, Neutro-3, EMT scores, and NET scores were highlighted by red triangles.


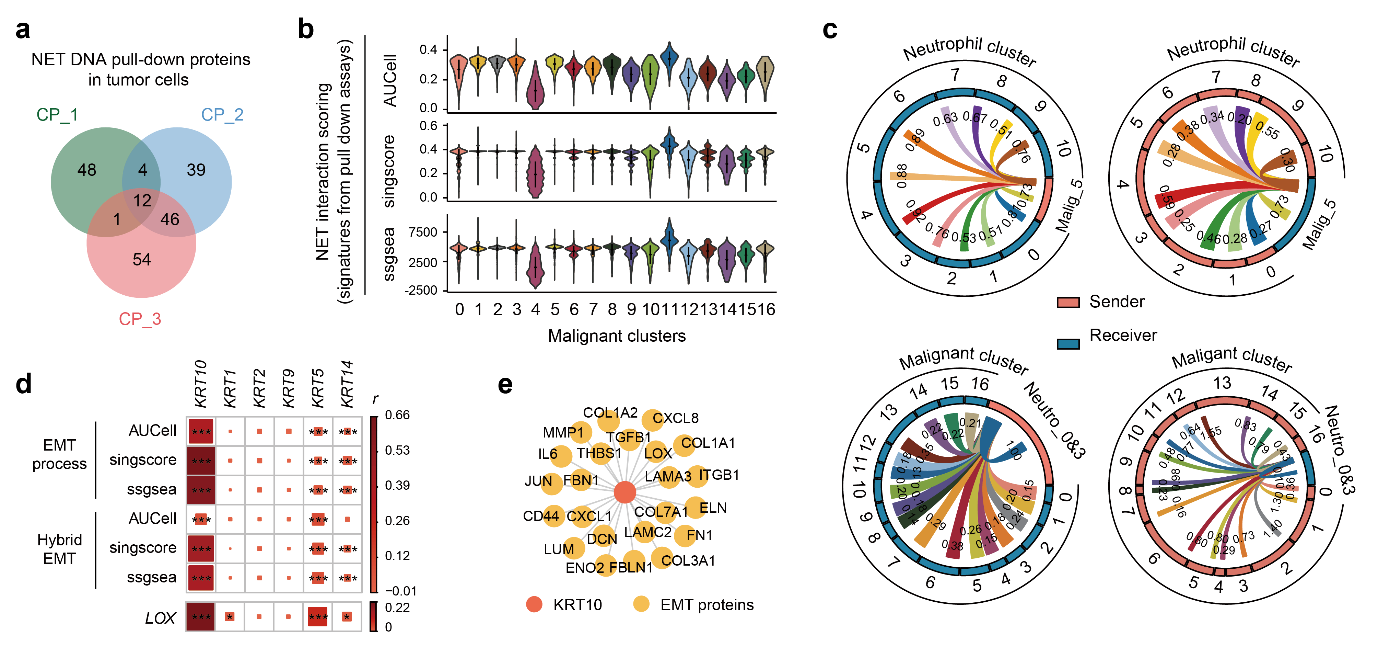


**Figure S10.** Neutro-0 and Neutro-3 interact with *LOX*^+^ Malig-5 through NET-KRT10. Related to Figure 4. a) Venn diagram depicting the overlap of NET DNA pull-down proteins in NSCLC cells. b) Violin plot showing NET binding scores (signatures extracted proteomic mass spectrometry following NET DNA pull-down assays), quantified by AUCell, singscore, and ssGSEA for each malignant cell subcluster in XY-NSCLC scRNA-seq data. c) Chord diagram showing cell–cell communication networks between neutrophil and malignant cell subclusters. d) Correlation of NET-binding protein expression with EMT scores and *LOX* expression in malignant cells. The scores were quantified by AUCell, singscore, and ssGSEA, and the correlations were analyzed using Spearman correlation. e) PPI network showing the interaction between KRT10 and EMT proteins. CP, Cytoplasmic protein.


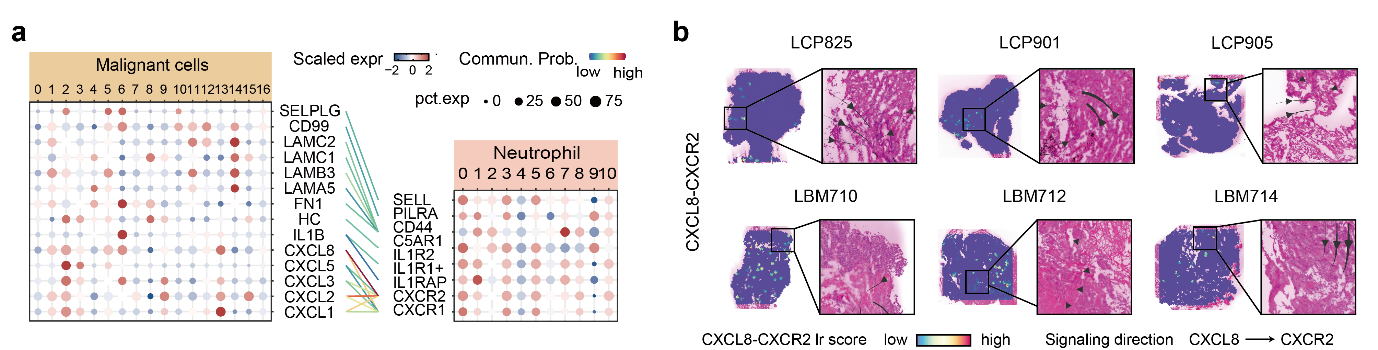


**Figure S11.** *LOX*^+^ Malig-5 interacts with Neutro-0 and Neutro-3 through CXCL8-CXCR2. a) Cell–cell communication plots of receptor-ligand pairs between malignant cell subclusters and neutrophil subclusters in the XY-NSCLC scRNA-seq data. b) Visualization of the spatial distribution of signaling scores and direction of CXCL8-CXCR2, using stLearn and COMMOT algorithms, on LCP (top) and LBM (bottom) spatial transcriptomics slides. Commun prob, ‌Communication probability‌; Expr, Expression; Lr, Ligand-receptor.

**
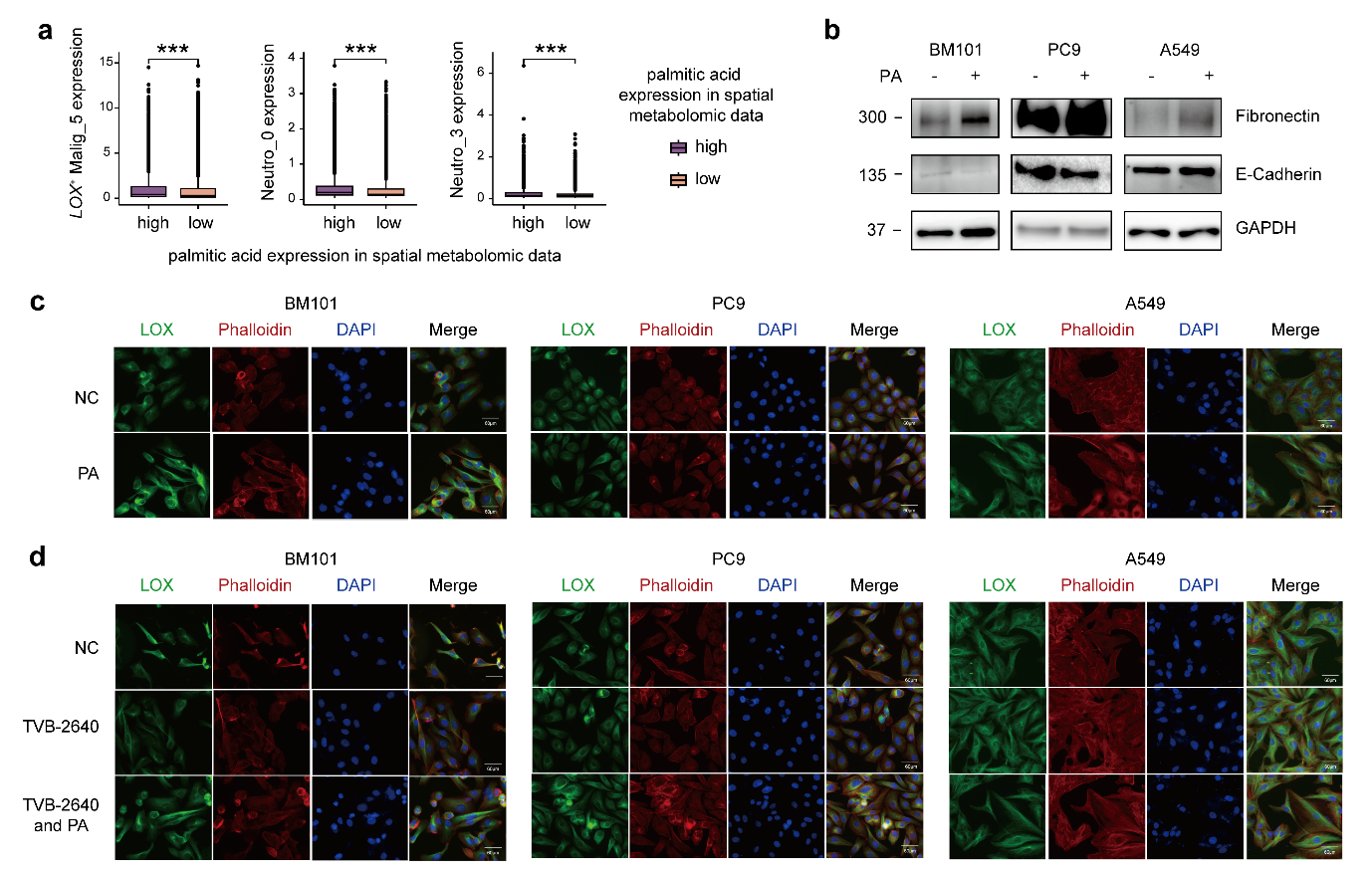
**

**Figure S12.** Palmitic acid is a potential key driver of the metastatic niche, and identifying therapeutic drugs that target palmitic acid to inhibit metastasis. Related to Figure 6. a) Comparison of *LOX*^+^ Malig-5, Neutro-0, and Neutro-3 proportions between high and low palmitic acid regions in the LCP and LBM spatial sequencing slides. The comparison was performed using two-tailed Wilcoxon test. *** *p* < 0.001. b) Western blot analysis of epithelial (E-cadherin) and mesenchymal (Fibronectin) markers in NSCLC cells (BM101, PC9, and A549) following 24-hour treatment with 50μM palmitic acid (PA). GAPDH was used as an internal loading control. c) Immunofluorescence staining of LOX (green), Phallodin (red) and DAPI (blue) in NSCLC cells (BM101, PC9, and A549) after 24-hour treatment with 50μM PA. Scale bars, 60 μm. d) Immunofluorescence staining of LOX (green), Phallodin (red) and DAPI (blue) in NSCLC cells (BM101, PC9, and A549) after 24-hour treatment with 0.1 μM TVB-2640 and with/without 50μM PA. Scale bars, 60 μm.


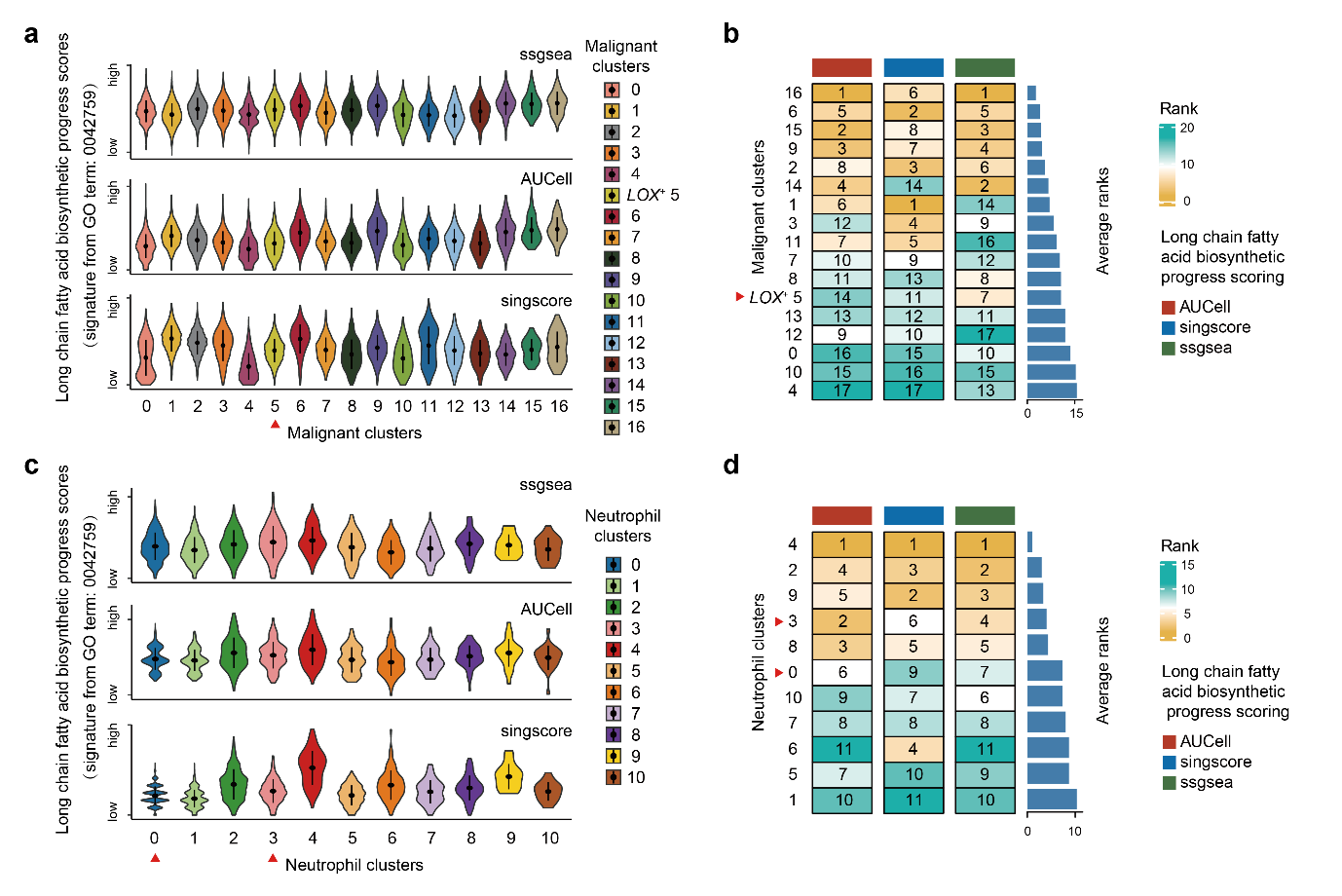


**Figure S13.** Quantification of long-chain fatty acid biosynthetic progress in neutrophil and malignant cell subpopulations. a) Violin plot of long-chain fatty acid biosynthetic progress (GO: 0042759) scores in XY-NSCLC malignant cell subcluster, quantified by ssGSEA (top), AUCell (middle), and singscore (bottom). b) Ranks of long-chain fatty acid biosynthetic progress (GO: 0042759) scores of malignant cell subclusters in each quantification algorithm of AUCell, singscore, and ssGSEA. c) Violin plot of long-chain fatty acid biosynthetic progress (GO: 0042759) scores in XY-NSCLC neutrophil subcluster, quantified by ssGSEA (top), AUCell (middle), and singscore (bottom). d) Ranks of long-chain fatty acid biosynthetic progress (GO: 0042759) scores of neutrophil subclusters in each quantification algorithm of AUCell, singscore, and ssGSEA. *LO^X^*^+^ Malig-5, Neutro-3, and Neutro-3 were highlighted by red triangles.


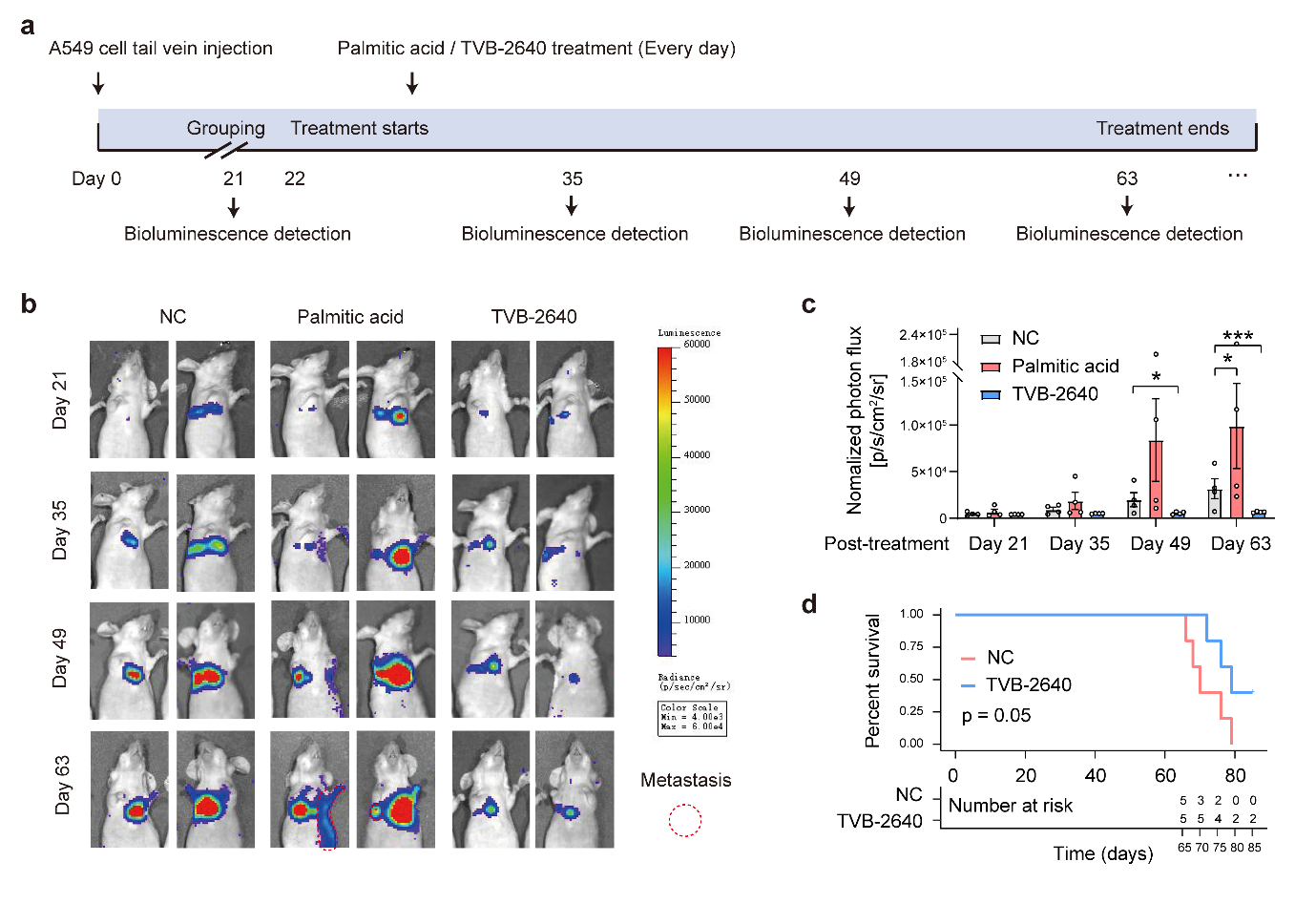


**Figure S14.** TVB-2640 suppresses metastasis and prolongs survival in NSCLC-bearing mice. a) Flowchart of the in vivo experiments of the A549 tail vein injection metastasis mouse model treated with palmitic acid or TVB-2640. The animal experiments were designed with two independent cohorts: one used for in vivo imaging and sample collection, and the other for assessing overall survival. b) Bioluminescence imaging of A549 tumor-bearing mice treated with palmitic acid, TVB-2640, or vehicle (NC) on days 21, 35, 49, and 63 after injection. Metastatic lesions were highlighted with red dashed circles. c) Comparison of A549 tumor growth in mice treated with palmitic acid, TVB-2640, or vehicle (NC), as assessed by normalized bioluminescent photon flux. *n* = 4 per group. Two-tailed Wilcoxon test. * *p* < 0.05, *** *p* < 0.001. d) Kaplan-Meier curves showing the cumulative survival probabilities of A549 tumor-bearing mice treated with vehicle (NC) or TVB-2640. *n* = 5 per group. Log-rank test.

**3. Tables**

**Table S1.** Feature summary of specimens and sequencing data for XY-NSCLC cohort

| Patient ID | Sample ID | Tissue origins | Histology | Pathology | Stages | Gene mutation | Age | Gender | Smoking | Preoperative treatments^*^ | No. of single cells | No. of spatial transcriptome spots | No. of spatial metabolome spots |
| --- | --- | --- | --- | --- | --- | --- | --- | --- | --- | --- | --- | --- | --- |
| PT1 | LCP721 | primary | LUAD & LUSC | MD | III | TTF-1, NapsinA, P53, CEA, P40 | 70 | Male | Never | No | 13999 | 3083 | 2482 |
| PT2 | LCP825 | primary | LUAD | MD | IA | TTF-1, NapsinA, CEA, CK7 | 75 | Male | Ex | No | 6141 | 2956 | 2482 |
| PT3 | LCP901 | primary | LUAD | MD | IIB | TTF-1, NapsinA, P53, CEA | 59 | Male | Ex | No | 9422 | 3315 | 2962 |
| PT4 | LCP905 | primary | LUAD | MD | IA | TTF-1, NapsinA, EVG, CD34 | 68 | Female | Never | No | 10443 | 3554 | 3051 |
| PT5 | LBM629 | BM | - | - | IV | EGFR, TTF-1, MOC31, P53, EMA | 65 | Male | Never | No | 11496 | - | - |
| PT6 | LBM705 | BM | LUAD | PD | IV | CK-Pan, TTF-1, NapsinA, | 59 | Male | Never | No | 9828 | 4156 | 4086 |
| PT7 | LBM710 | BM | LUAD | MD | IV | TTF-1, NapsinA, P53, EGFR, HER2, MSH2, MSH6, LH1, PMS2, CK67, CK19 | 71 | Female | Never | No | 8455 | 2904 | 2580 |
| PT8 | LBM712 | BM | LUAD | PD | IV | CK-Pan, TTF-1, NapsinA | 54 | Male | Never | No | 13123 | 4477 | 3727 |
| PT9 | LBM714 | BM | LUAD | - | IV | CK-Pan, EMA, TTF-1, NapsinA, CK7 | 32 | Female | Never | No | 11476 | 4059 | 3348 |

^*^ Preoperative treatments include radiotherapy, chemotherapy, TKI, and immunotherapy.

Abbreviation‌: Tissue origins (BM, brain metastasis); Histology (LUAD, Lung adenocarcinoma; LUSC, Lung Squamous Cell Carcinoma); Pathology (MD, Moderately differentiated; PD, Poorly differentiated); Smoking (Ex, Ex-smoker; Never, Never smoker).

**4. Reference**

1. Patel, A.P., Tirosh, I., Trombetta, J.J., Shalek, A.K., Gillespie, S.M., Wakimoto, H., Cahill, D.P., Nahed, B.V., Curry, W.T., Martuza, R.L., et al. (2014). Single-cell RNA-seq highlights intratumoral heterogeneity in primary glioblastoma. Science *344*, 1396-1401. 10.1126/science.1254257.

2. Sun, H.F., Li, L.D., Lao, I.W., Li, X., Xu, B.J., Cao, Y.Q., and Jin, W. (2022). Single-cell RNA sequencing reveals cellular and molecular reprograming landscape of gliomas and lung cancer brain metastases. Clin Transl Med *12*, e1101. 10.1002/ctm2.1101.

3. Zhang, L., Li, Z., Skrzypczynska, K.M., Fang, Q., Zhang, W., O'Brien, S.A., He, Y., Wang, L., Zhang, Q., Kim, A., et al. (2020). Single-Cell Analyses Inform Mechanisms of Myeloid-Targeted Therapies in Colon Cancer. Cell *181*, 442-459.e429. 10.1016/j.cell.2020.03.048.

4. Suo, S., Zhu, Q., Saadatpour, A., Fei, L., Guo, G., and Yuan, G.C. (2018). Revealing the Critical Regulators of Cell Identity in the Mouse Cell Atlas. Cell Rep *25*, 1436-1445.e1433. 10.1016/j.celrep.2018.10.045.

5. Liberzon, A., Birger, C., Thorvaldsdóttir, H., Ghandi, M., Mesirov, J.P., and Tamayo, P. (2015). The Molecular Signatures Database (MSigDB) hallmark gene set collection. Cell Syst *1*, 417-425. 10.1016/j.cels.2015.12.004.

6. Malagoli Tagliazucchi, G., Wiecek, A.J., Withnell, E., and Secrier, M. (2023). Genomic and microenvironmental heterogeneity shaping epithelial-to-mesenchymal trajectories in cancer. Nat Commun *14*, 789. 10.1038/s41467-023-36439-7.

7. Xu, J., Cao, Y., Wu, X., Cheng, Y., and Zhang, X. (2023). Neutrophil extracellular traps-mediated molecular subtypes characterize the hallmarks of tumor microenvironment and guide precision medicine in hepatocellular carcinoma. Journal of Radiation Research and Applied Sciences *16*, 100577. <https://doi.org/10.1016/j.jrras.2023.100577>.

8. Xiao, Z., Dai, Z., and Locasale, J.W. (2019). Metabolic landscape of the tumor microenvironment at single cell resolution. Nat Commun *10*, 3763. 10.1038/s41467-019-11738-0.

9. Chen, D.S., and Mellman, I. (2013). Oncology meets immunology: the cancer-immunity cycle. Immunity *39*, 1-10. 10.1016/j.immuni.2013.07.012.

10. Xu, L., Deng, C., Pang, B., Zhang, X., Liu, W., Liao, G., Yuan, H., Cheng, P., Li, F., Long, Z., et al. (2018). TIP: A Web Server for Resolving Tumor Immunophenotype Profiling. Cancer Res *78*, 6575-6580. 10.1158/0008-5472.Can-18-0689.

11. Thorsson, V., Gibbs, D.L., Brown, S.D., Wolf, D., Bortone, D.S., Ou Yang, T.H., Porta-Pardo, E., Gao, G.F., Plaisier, C.L., Eddy, J.A., et al. (2018). The Immune Landscape of Cancer. Immunity *48*, 812-830.e814. 10.1016/j.immuni.2018.03.023.

12. Bäckdahl, J., Franzén, L., Massier, L., Li, Q., Jalkanen, J., Gao, H., Andersson, A., Bhalla, N., Thorell, A., Rydén, M., et al. (2021). Spatial mapping reveals human adipocyte subpopulations with distinct sensitivities to insulin. Cell Metab *33*, 1869-1882.e1866. 10.1016/j.cmet.2021.07.018.

13. Xie, Y., Zhang, L., Wang, L., Chen, B., Guo, X., Yang, Y., Shi, W., Chen, A., Yi, J., Tang, J., and Xiang, J. (2024). EphB1 promotes the differentiation and maturation of dendritic cells in non-small cell lung cancer. Cancer Lett *582*, 216567. 10.1016/j.canlet.2023.216567.
